# Identification and experimental validation of diagnostic and prognostic genes CX3CR1, PID1 and PTGDS in sepsis and ARDS using bulk and single-cell transcriptomic analysis and machine learning

**DOI:** 10.3389/fimmu.2024.1480542

**Published:** 2024-12-23

**Authors:** Jijin Jiang, Yan Chen, Yue Su, Li Zhang, Hao Qian, Xinmiao Song, Jin-Fu Xu

**Affiliations:** ^1^ Department of Respiratory and Critical Care Medicine, Shanghai Pulmonary Hospital, School of Medicine, Tongji University, Shanghai, China; ^2^ Institute of Respiratory Medicine, School of Medicine, Tongji University, Shanghai, China

**Keywords:** sepsis, ARDS, machine learning, single-cell, diagnosis, prognosis

## Abstract

**Background:**

Sepsis is an uncontrolled reaction to infection that causes severe organ dysfunction and is a primary cause of ARDS. Patients suffering both sepsis and ARDS have a poor prognosis and high mortality. However, the mechanisms behind their simultaneous occurrence are unclear.

**Methods:**

We acquired sepsis and ARDS datasets from GEO and Arrayexpress databases and screened hub genes by WGCNA and machine learning algorithm. For diagnosis and prognosis, ROC curve and survival analysis were used. We performed GO, KEGG, GSEA, immune cell infiltration, drug prediction, molecular docking, transcription factor prediction, and constructed PPI and ceRNA networks to explore these genes and the common mechanisms of sepsis and ARDS. Single-cell data analysis compared immune cell profiles and hub gene localization. Finally, RT-qPCR and H&E staining confirmed the reliability of hub genes using PBMCs samples and mouse models.

**Results:**

We identified 242 common differentially expressed genes in sepsis and ARDS. WGCNA analysis showed that the turquoise module in GSE95233 is strongly linked to sepsis occurrence and poor prognosis, while the black module in GSE10474 is associated with ARDS. Using WGCNA and three machine learning methods (LASSO, random forest and Boruta), we identified three key genes CX3CR1, PID1 and PTGDS. Models built with them showed high AUC values in ROC curve evaluations and were validated by external datasets, accurately predicting the occurrence and mortality. We further explored the immunological landscape of these genes using immune infiltration and single-cell analysis. Then, the ceRNA, predicted drugs and molecular docking were analyzed. Ultimately, we demonstrated that these genes are expressed differently in human and mouse samples with sepsis and ARDS.

**Conclusion:**

This study identified three molecular signatures (CX3CR1, PID1 and PTGDS) linked to the diagnosis and poor prognosis of sepsis and ARDS, validated by RT-qPCR and H&E staining in both patient and mouse samples. This research may be valuable for identifying shared biological mechanisms and potential treatment targets for both diseases.

## 1 Introduction

Sepsis is characterized as an abnormal host response to infection that can result in multiorgan failure, acidosis and death (1, 2). Data show that the in-hospital mortality rate of sepsis is 17%-26%, accounting for 36.9%-55.9% of all in-hospital deaths in the United States (3, 4). Hospitalizations for sepsis have increased dramatically over the past decade (5). Sepsis not only manifests as increased inflammation, but also immunosuppression, leading to cellular dysfunction and systemic organ failure, including the respiratory system, cardiovascular system, nervous system (6), etc.

ARDS, or acute respiratory distress syndrome, is a frequently occurring clinical complication of sepsis. It is marked by the sudden development of low blood oxygen levels, noncardiogenic pulmonary edema, low blood oxygen levels, and the necessity for mechanical breathing support (7). Sepsis is a leading cause of ARDS, accounting for approximately 75% of cases (8). Sepsis-induced ARDS is associated with higher severity of illness, lower success rates of extubation, and increased mortality compared to ARDS not caused by sepsis (9). There are still challenges in the individualized treatment of ARDS. Although the implementation of lung-protective ventilation, strict fluid management, and the application of extracorporeal membrane oxygenation (ECMO) have improved ARDS symptoms, the mortality rate for ARDS remains high at 40% (10, 11). The extremely high mortality rate of sepsis and ARDS has brought a huge burden to human health and socioeconomic development (12, 13). At present, the relationship between sepsis and ARDS is not completely clear, and the common diagnostic targets for sepsis and ARDS remain under-researched. There is substantial evidence that early detection of sepsis and organ failure reduces severity and ultimately mortality (14, 15). Therefore, it is an urgent need for biomarkers that can accurately identify the occurrence of sepsis and ARDS and predict their poor prognosis (15–17).

Biomarkers are essential for early diagnosis, detecting organ dysfunction, and improving prognosis. Current sepsis biomarkers include acute phase proteins, cytokines, and cellular markers, but their performance remains suboptimal (18, 19). ARDS diagnosis mostly relies on clinical symptoms, leading to missed or delayed cases (20). Recent studies identify SCAMP5, NDUFB3, and LILRA5 as potential sepsis biomarkers, with LPIN1, CYBB and FCAR linked to prognosis (21–25). For ARDS, TYMS and STAT3 may assist in diagnosis, and LCN2 could predict survival (26–28).

However, few studies have explored biomarkers that can simultaneously predict both the diagnosis and poor prognosis of sepsis and ARDS. Although sepsis-induced ARDS has been investigated as a single illness (29, 30), the relationship between sepsis and ARDS is not well understood. There is limited research into their shared regulatory mechanisms, despite these two conditions being prevalent and difficult to manage in clinical practice. The novelty of our study lies in the identification of three genes that are not only associated with the diagnosis of both sepsis and ARDS but are also predictive of poor patient outcomes. Our research clearly demonstrates the existence of common molecular targets between these two diseases, highlighting a potential shared pathway for therapeutic intervention. This provides valuable insight into the overlapping mechanisms of sepsis and ARDS, offering a foundation for future studies aimed at improving diagnostic and treatment strategies for both conditions.

Our study identified shared indicators of ARDS and sepsis that predict diagnosis and prognosis. Using bioinformatics techniques and machine learning models, we analyzed data from GEO and ArrayExpress, pinpointing the diagnostic genes CX3CR1, PID1, and PTGDS, which predict poor prognosis. We conducted gene set enrichment analysis, compared immune cell differences via single-cell sequencing, and explored potential treatments through molecular docking. Validation was performed using PBMCs from healthy volunteers and patients, as well as mouse samples. Our findings provide insights into the mechanisms of ARDS and sepsis, highlighting key genes and therapeutic targets for early diagnosis and prognosis.

## 2 Materials and methods

### 2.1 Data collection and processing

**Figure 1:**
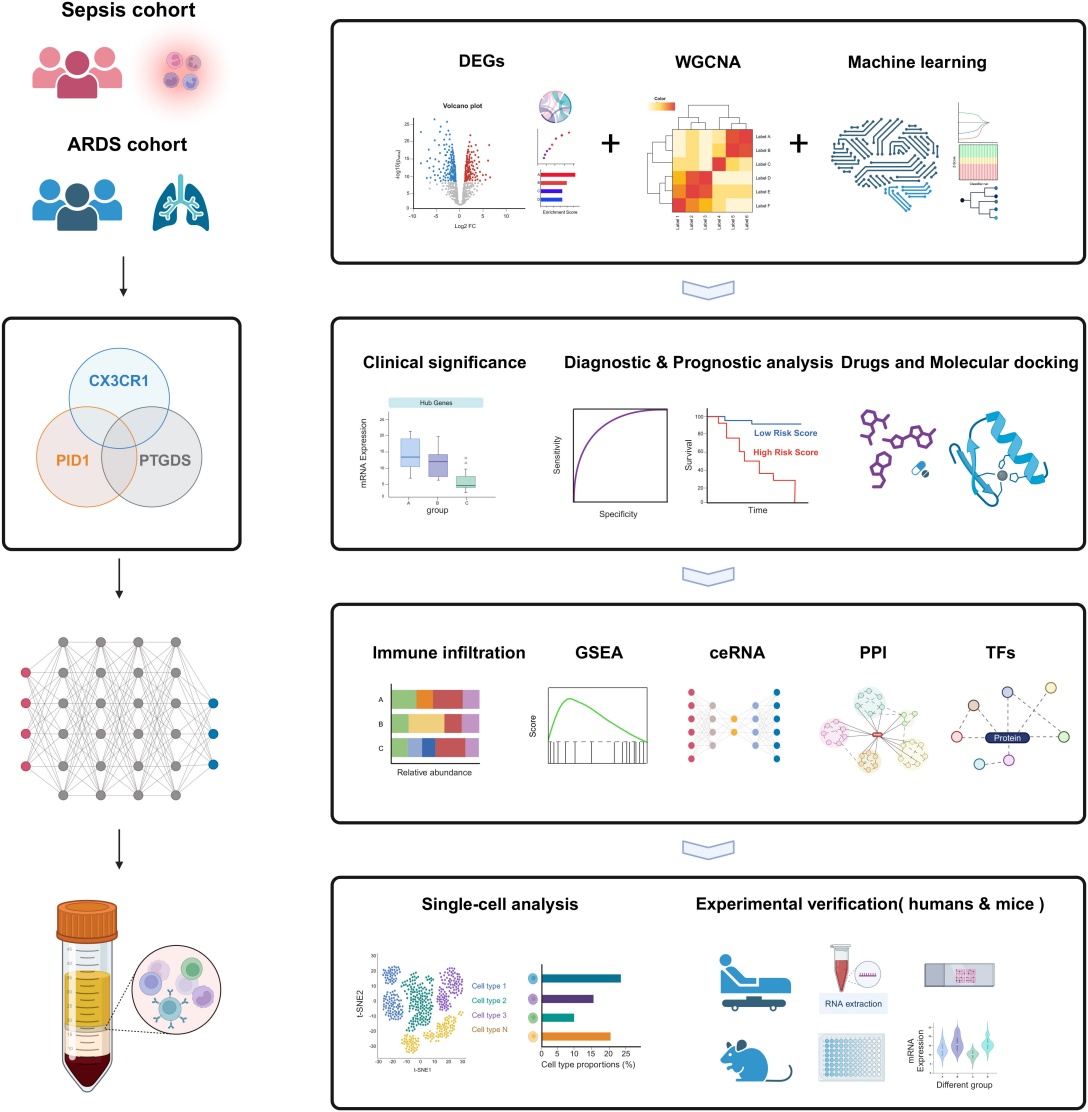
The flow chart for the whole design. Created with BioRender.com.

The peripheral blood expression profile was downloaded from GEO (http://www.ncbi.nlm.nih.gov/geo) and ArrayExpress (https://www.ebi.ac.uk/arrayexpress/) database. We have retrospectively enrolled 11 datasets. The dataset search criteria included: 1) experimental data types such as microarray or high-throughput sequencing; 2) patient samples collected within 48 hours of admission; 3) human blood samples; 4) studies concentrating on sepsis or ARDS; 5) datasets from the same sequencing platform, producing two separate expression profiles. **Table 1** offers a brief overview of the comprehensive details of these datasets. The datasets GSE65682, GSE95233, and GSE167363 served as discovery cohorts for sepsis, while GSE32707, GSE10474, and GSE151263 were utilized as discovery cohorts for ARDS. Furthermore, datasets GSE57065, GSE54514, GSE13904, E-MTAB-5273, and E-MTAB-5274 served as validation groups for sepsis and ARDS, respectively. The essential steps carried out in this article are illustrated in the diagram (**Figure 1**).

**Table 1:** Details of GEO datasets used in the study.

Disease	GSE number	Source type	Samples size	Samples size	GPL Platform	Group
Sepsis	GSE65682	Whole blood	Healthy people(n=42)	Sepsis patients(n=760)	GPL13667	Discovery cohort
	GSE95233		Healthy people(n=22)	Sepsis patients(n=102)	GPL570	Discovery cohort
	GSE57065		Healthy people(n=25)	Sepsis patients(n=82)	GPL570	Validation cohort
	GSE54514		Healthy people(n=36)	Sepsis patients(n=127)	GPL6947	Validation cohort
	GSE13904		Healthy people(n=18)	Sepsis patients(n=158)	GPL570	Validation cohort
	GSE185263		Healthy people(n=44)	Sepsis patients(n=348)	GPL16791	Validation cohort
	GSE63311		Healthy people(n=11)	Sepsis patients(n=37)	GPL10999	Validation cohort
	GSE48080	PBMC	Healthy people(n=3)	Sepsis patients(n=20)	GPL4133	Validation cohort
	GSE167363	PBMC	Healthy people(n=2)	Sepsis patients(n=4)	GPL24676	Discovery cohort
ARDS	GSE32707	Whole blood	Sepsis patients(n=58)	Sepsis+ARDS patients(n=31)	GPL10558	Discovery cohort
	GSE10474		Sepsis patients(n=21)	Sepsis+ARDS patients(n=13)	GPL571	Discovery cohort
	E-MTAB-5273		Healthy people(n=10)	Sepsis+ARDS patients(n=127)	A-MEXP-2210	Validation cohort
	E-MTAB-5274		Healthy people(n=10)	Sepsis+ARDS patients(n=42)	A-MEXP-2210	Validation cohort
	GSE151263	PBMC	Sepsis patients(n=2)	Sepsis+ARDS patients(n=2)	GPL20301	Discovery cohort

To ensure reliable analysis, we standardized datasets prior to processing. First, we applied median normalization by subtracting the median log ratio of each chip, ensuring a median log ratio of 0 across all chips for comparability. Next, we filtered out low-expressing genes, selecting those expressed in at least 75% of samples. Gene expression values were log2-transformed to normalize the data, which reduces the impact of extreme values and makes it suitable for statistical analysis. To account for batch effects, we applied uniform criteria for differential gene screening across datasets, selecting genes with a P-value < 0.05 and an absolute fold change > 0.3. Finally, we used a Venn diagram to identify common differentially expressed genes across datasets, improving the robustness of the results (31, 32).

### 2.2 Differentially expressed gene identification

The LIMMA package in R (version 3.58.1) was employed to discover the genes with differential expression between the two groups. DEGs were identified as genes meeting the criteria of a P-value below 0.05 and an absolute fold change exceeding 0.3 relative to the control. The ‘ggplot2’ package was utilized to create volcano plots for differentially expressed genes in both sepsis and ARDS groups. The ‘VennDiagram’ package was utilized to identify the DEGs common to both sepsis and ARDS.

### 2.3 Functional analysis of DEGs

To explore the biological processes of the key genes connecting sepsis and ARDS, functional enrichment studies were conducted. The Omicshare web tool (https://www.omicshare.com/tools) was employed for gene ontology (GO) annotation to examine the functional annotation of DEGs. The identified DEGs were analyzed for pathways using the online Omicshare tool, referencing the KEGG pathway database. Annotation terms with a P value below 0.05 were considered significantly enriched, and the results were visualized through bubble charts, bar graphs, and cycle diagrams.

### 2.4 Building the network for weighted gene co-expression network analysis

Focusing on the most variable 25% of genes, we employed the WGCNA package to identify the co-expression network and selecting genes from various clusters. The pickSoftThreshold function was employed to identify the soft threshold power (b), ensuring it met the scale-free topology criterion. Subsequently, gene co-expression clusters were determined using a single-step network building method. Every module included a minimum of 30 genes, and any leftover genes that were not grouped were placed in the grey module. Afterward, we calculated the correlation coefficients between the modules and phenotypes to determine which modules were closely associated with sepsis or ARDS. For ARDS, we selected the black module, which had the highest correlation, and conducted a further analysis of the associated genes. Nevertheless, several modules had a substantial association with sepsis, prompting us to use gene significance (GS) and module membership (MM) as filters for the genes. Subsequently, we selected genes within the hub modules that exhibited a |MM| value more than 0.6 and a |GS| value greater than 0.6 specifically for the sepsis group.

### 2.5 Screening hub genes by machine learning

To refine the selection of potential genes for diagnosing sepsis and ARDS, three different machine learning techniques were utilized. The first method, called Least Absolute Shrinkage and Selection Operator (LASSO) regression, improves prediction accuracy by choosing variables and using regularization, thereby refining statistical models (33). The second method, random forest (RF), excels because it imposes no restrictions on variable conditions and offers high levels of accuracy, sensitivity, and specificity. Random Forests are capable of predicting continuous values and offering stable forecasts (34). The Boruta algorithm, which is the third method, selects features by randomly shuffling each actual feature, evaluating their significance, and progressively eliminating those with low correlation to pinpoint the most pertinent variables (35). This research employed the Boruta package (v8.0.0) for feature selection, building 500 trees to assess the diagnostic significance of these biomarkers for various diseases. The R packages “glmnet”, “randomForest”, and “Boruta” were employed to conduct the machine learning analysis. The intersection of genes identified by LASSO regression, WGCNA, RF and Boruta were considered as candidate hub genes for diagnosing sepsis and ARDS.

### 2.6 Nomogram establishment and assessment

Nomograms are effective instruments for combining various indicators to forecast the onset and advancement of diseases. In our research, we created a nomogram model that uses essential genetic indicators to forecast the onset of sepsis and ARDS, employing the ‘rms’ package in R. We used the ‘pROC’ R package to perform a receiver operating characteristics (ROC) analysis, measuring the model’s sensitivity and accuracy to evaluate its performance. The genetic characteristics were confirmed with a separate validation group. To confirm the nomogram’s predictive precision, we utilized calibration curves and performed validation on the test set. Additionally, we employed the ‘ggDCA’ R package to perform decision curve analysis (DCA) for the genetic signature as well as the nomogram model. This extensive analysis aimed to evaluate the clinical relevance of the model and explore its potential applications in real-world medical settings.

### 2.7 Construction of the prognostic prediction model by hub genes

At the start of the survival research, patients were classified as having high or low hub gene expression based on the best threshold value. Kaplan-Meier plots were created to determine the relationship between the expression of three critical genes and overall survival.

Secondly, key genes pinpointed via univariate Cox regression were chosen as markers for subsequent multivariate Cox regression evaluation. Using the ‘survival’ R package to calculate the median risk score, we compared the low-risk and high-risk groups. The ROC curve was generated with the ‘survivalROC’ package to assess the prediction model’s sensitivity and specificity. The predictive model’s effectiveness was evaluated by the area under the ROC curve (AUC), where a score between 0.5 and 0.7 denotes moderate performance, 0.7 to 0.8 indicates good accuracy, and anything above 0.9 signifies outstanding results. We create a predictive tool using essential genes from the prognostic framework and patient clinical data to estimate the 28-day mortality risk in individuals with sepsis.

### 2.8 Immune infiltration analysis

CIBERSORT analysis was performed on each disease sample to assess the proportions of various immune cells. The CIBERSORT method determines the makeup of immune cells by analyzing gene expression profiles through deconvolution. LM22 has 22 known gene signatures, according to the CIBERSORT website (http://cibersort.stanford.edu/). We assessed 22 unique immune cell types based on the LM22 gene signature using CIBERSORT and 1000 iterations. We selected samples for the next analysis whose CIBERSORT P value was less than 0.05. In order to facilitate comparisons between immune cell types and datasets, the output estimates from CIBERSORT were adjusted to sum to one for each sample. The results were visualized using the R packages ‘corrplot,’ ‘vioplot,’ and ‘ggplot2’. Additionally, the relationship between immune cell infiltration and diagnostic biomarkers was assessed using Spearman’s nonparametric correlation.

### 2.9 Gene set enrichment analysis

Initially, the samples in the dataset were classified as either high or low expression groups based on the expression level of a single key gene. Afterwards, the ‘clusterProfiler’ software was used for GSEA in order to identify the pathways associated with the important genes. An enrichment analysis was performed with a p-value cutoff of below 0.05. Enrichplot was employed to display the top five pathways that either activate or inhibit each gene within the two disease categories.

### 2.10 Competing endogenous RNA

A ceRNA acts as a competitive binding agent for RNA molecules. A ceRNA regulatory network includes the entire set of interactions among ceRNAs, generally involving mRNA, miRNAs, and lncRNAs. We explored miRNAs that associate with hub genes using the databases miRDB (https://mirdb.org/), miRabel (http://bioinfo.univ-rouen.fr/mirabel/), miRWalk (http://mirwalk.umm.uni-heidelberg.de/), and Targetscan (https://www.targetscan.org/vert_80/). Afterward, we employed the lncBase database to find lncRNAs predicted to regulate various miRNAs. The alluvial plot of ceRNA was created using the OmicShare web-based tools.

### 2.11 Transcriptional regulatory analysis

The Cistrome Data Browser (Cistrome DB) is a specialized data portal for chromatin profiling methods (36, 37). This study explored the relationship between significant genes and TFs using the Cistrome database. The reference genome was aligned to hg38, with the transcription start point positioned at 10 kilobases. The transcription factor regulatory network was displayed using Cytoscape (38).

### 2.12 Analysis of protein interaction networks

The analysis of DEGs for protein-protein interactions (PPIs) utilized the STRING database (version 12.0). Interactions with an aggregate score exceeding 0.4 were deemed statistically meaningful. Pictures sourced from STRING were altered with Cytoscape, and the MCODE extension identified key interacting genes. For further analysis, all genes capable of interacting within the PPI network were chosen.

### 2.13 Screening of small molecule compounds and analysis of molecular-ligand interactions through docking

The CMap database (https://clue.io/) is a resource for predicting drugs based on differential gene expression, mainly utilized to investigate the functional links between genes, small molecules, and diseases. The CMap database conducts transcriptome analysis on cell lines treated with various compounds and investigates the alterations in gene expression relative to untreated controls. The expression patterns of differentially expressed genes in sepsis and ARDS patients were uploaded to the CMap database. Substances with low enrichment scores could potentially alter the expression patterns of the uploaded gene data. After that, enrichment score ranking selected the top 10 small molecule compounds that potentially change uploaded gene expression. These compounds might have the capacity to avert the development of ARDS and sepsis in patients. Furthermore, we implemented Enrichr (https://maayanlab.cloud/Enrichr/) to identify potential molecular compounds could potentially influence critical genes. Enrichr offers numerous gene set libraries for analysis, enabling genome-wide gene-set enrichment exploration.

We prioritized four small molecules that were suggested in the literature to be active in inflammation-related biological processes for molecular docking, and the results showed that they have the potential to be further developed into drugs. Above all, small molecule ligand two-dimensional structures from PubChem were used for molecular-ligand docking investigations. Subsequently, Chem Office 20.0 software was utilized to convert these into 3D models, which were then stored in mol2 format. High-resolution protein target crystal structures from the RCSB PDB library (http://www.rcsb.org/) were chosen as molecular receptors. With PyMOL 2.6.0, the protein structures underwent refinement by eliminating water molecules and phosphate groups, and thereafter were stored as PDB files. MOE 2019 software facilitated energy minimization of the compounds, prepared the target proteins, and pinpointed active sites. Finally, the process of molecular docking was carried out using MOE 2019, with a total of 50 iterations. PyMOL 2.6.0 and Discovery Studio 2019 software were used to visualize the results of binding energy calculations, which were used to determine the binding affinity.

### 2.14 ScRNA-seq data collection

The scRNA-seq dataset GSE167363, available in the GEO database, contains single-cell RNA sequencing information of PBMCs from healthy people, sepsis survivors, and those who did not survive gram-negative sepsis. The dataset comprised six samples: GSM5102900 (HC1, PBMCs from a healthy individual), GSM5102901 (HC2, PBMCs from another healthy individual), GSM5102903 (NS1, PBMCs from a non-survivor), GSM5511352 (NS2, PBMCs from another non-survivor), GSM5511354 (S1, PBMCs from a survivor), and GSM5511356 (S2, PBMCs from another survivor).

The GEO repository supplied the single-cell RNA sequencing dataset GSE151263, containing scRNA-seq data of PBMCs from individuals with sepsis and those with sepsis complicated by ARDS. A total of six samples were examined: GSM4569780 (S1, PBMCs from sepsis patients), GSM4569781 (S2, PBMCs from sepsis patients), GSM4569785 (NS1, PBMCs from non-survivors), GSM5511352 (SA1, PBMCs from non-survivors), GSM5511354 (S1, PBMCs from patients with sepsis and ARDS), and GSM4569786 (SA2, PBMCs from patients with sepsis and ARDS).

### 2.15 scRNA-seq data processing and analysis

To control for batch effects, we implemented the following steps: 1) Gene and Cell Filtering: We filtered the data using parameters of min.cells = 3 and min.features = 200, ensuring each cell expressed at least 200 genes and each gene was expressed in at least 3 cells. This reduced the impact of low-quality data; 2) Normalization: The gene expression matrix was standardized using the “NormalizeData” and “FindVariableFeatures” functions. “NormalizeData” ensured consistent expression levels, while “FindVariableFeatures” identified the top 2000 highly variable genes (HVGs) for further analysis; 3) Controlling Confounding Factors: Using the “ScaleData” function, we standardized HVG expression while accounting for mitochondrial and hemoglobin genes (“percent.mt,” “percent.HB”), minimizing their influence as confounding factors. This function also applied z-score normalization to the HVG matrix; 4) Batch Effect Removal: The “RunHarmony” function was used to correct for batch effects, integrating datasets to ensure a uniform distribution of cells with similar characteristics across batches; 5) Dimensionality Reduction: After Harmony correction, we applied UMAP via the “RunUMAP” function for dimensionality reduction, visualizing cell distribution and confirming batch effect removal.

Before data integration, we preprocessed raw data by filtering cells, retaining those that expressed at least 200 genes and genes expressed in at least 3 cells, mitigating low-quality data. Seurat objects were created, and the expression data was normalized, identifying the top 2000 HVGs. We also accounted for mitochondrial and hemoglobin gene proportions using the “ScaleData” function. After merging datasets, the “RunHarmony” function was employed for batch correction, followed by principal component analysis (PCA) for feature selection. Dimensionality reduction was performed using UMAP and t-SNE for visualization. Clustering was carried out using the “FindNeighbors” and “FindClusters” functions, with clusters visualized through UMAP plots. Marker genes were identified using “FindAllMarkers,” and cell types were annotated based on clustering results, followed by cell proportion analysis and further visualizations.

### 2.16 Isolation of PBMCs, RNA extraction and RT-qPCR analysis

Patients who were eligible for sepsis (n=16) or sepsis-induced ARDS (n=15) within 24 hours in the intensive care unit of Shanghai Pulmonary Hospital were included in this study. Patients met the following criteria: 1) Patients aged 18 years or older; 2). The Third International Consensus Definitions for Sepsis (Sepsis-3) were used to diagnose sepsis (2). 3) Patients diagnosed with ARDS patients who met the ARDS criteria updated by the European Society of Intensive Care Medicine (ESICM) in July 2023 (39). Our study was approved by the Research Ethics Committee of Our study was approved by the Research Ethics Committee of Shanghai Lung Hospital (No. Q24-410), and informed consent was obtained from all participants. A table of the clinical characteristics of our collected patients is shown in **Table 2**.

**Table 2:** Baseline characteristics of patients with sepsis and ARDS.

Characteristic	Sepsis (n=16)	ARDS (n=15)
Age (years)	70.25 (7.64)	70.40 (7.88)
Sex [male (%)]	9 (56.25)	8 (53.33)
White blood cell count (*10^9/L)	13.280 [9.0,20.2]	10.800 [8.8,20.8]
Neutrophil percentage (%)	86.000 [76.7,91.6]	87.000 [75.4,91.9]
Lactate (mmol/L)	2.840 [2.0,3.2]	2.970 [2.7,3.3]
PCT (ng/ml)	1.015 [0.2,3.2]	1.510 [0.2,3.4]
D-dimer (mg/ml)	2.563 [1.6,4.7]	2.526 [1.6,4.8]
Blood Sugar (mmol/L)	9.900 [7.5,12.5]	9.300 [7.4,12.0]
Comorbidities [n] (%)		
Chronic pulmonary disease	11 (68.75)	10 (66.67)
Chronic kidney disease	2 (12.50)	0 (0.00)
Heart disease	9 (56.25)	9 (60.00)
Hepatic disease	8 (50.00)	7 (46.67)
Gastrointestinal disease	5 (31.25)	5 (33.33)
Diabetes mellitus	4 (25.00)	3 (20.00)
Focus of infection [n] (%)		
Pulmonary	14 (87.50)	13 (86.67)
Urinary tract	3 (18.75)	0 (0.00)
Gastrointestinal tract	2 (12.50)	0 (0.00)
Grading of severity (%)		
mild		0 (0.00)
moderate		8 (53.33)
severe		7 (46.67)
SOFA score	4.000 [3.0,4.8]	3.000 [2.0,5.0]
Length of ICU stay (days)	12.500 [10.0,31.0]	14.000 [10.0,33.0]
Mechanical ventilation time (days)	9.500 [0.0,15.5]	10.000 [8.0,16.0]
Mortality (%)	8 (50.00)	9 (60.00)

Data are presented as mean (standard deviation), n (%), or median [First quartile, Third quartile].

We collected blood of patients or controls and diluted with PBS (Servicebio, Wuhan, China). Subsequently, we meticulously applied blood onto the Lymphoprep density gradient medium provided by STEMCELL Technologies, located in Vancouver, BC, Canada. Following a 30-minute centrifugation at 700g, the PBMCs layer located in the middle of the solution was gently removed. After resuspending PBMCs in PBS, we added Red Blood Cell lysis buffer (Beyotime, Shanghai, China) and let it stand at room temperature for 10 minutes. We acquire the PBMCs by centrifuging at 300g for five minutes.

Total RNA was extracted from PBMCs using TRIzol reagent (Invitrogen, Carlsbad, California, USA). Subsequently, RNA was transformed into cDNA using the Hifair III First Strand cDNA Synthesis SuperMix for qPCR (Yeasen, Shanghai, China). Yeasen’s Hieff qPCR SYBR^®^ Green Master Mix (Shanghai, China) was employed to perform real-time quantitative PCR (RT-qPCR). The 2-DDct technique was employed to determine relative gene expression levels. GAPDH was utilized as a control benchmark. Each experiment was conducted three times. The RT-qPCR primers for persons are as follows: GAPDH forward GGAGCGAGATCCCTCCAAAAT, reverse GGCTGTTGTCATACTTCTCATGG; CX3CR1 forward AGTGTCACCGACATTTACCTCC, reverse AAGGCGGTAGTGAATTTGCAC; PID1 forward TCCTGGAAATCCGGCCATTC, reverse AGGTCATCATTGATCTCCCTGT; PTGDS forward GGCGTTGTCCATGTGCAAG, reverse GGACTCCGGTAGCTGTAGGA. The RT-qPCR primers for mice are as follows: GAPDH forward AGGTCGGTGTGAACGGATTTG, reverse GGGGTCGTTGATGGCAACA; CX3CR1 forward GAGTATGACGATTCTGCTGAGG, reverse CAGACCGAACGTGAAGACGAG; PID1 forward AAGGAACCTATACCAGCAGTCC, reverse CCTTGCAGCCACTATGAGTCC; PTGDS forward GAAGGCGGCCTCAATCTCAC, reverse CGTACTCGTCATAGTTGGCCTC.

### 2.17 Rodent sepsis model caused by cecal ligation and puncture

Eight to ten-week-old, pathogen-free C57BL/6 male mice in good health were obtained from Shanghai Slack Laboratory Animal Co., LTD [license number: SExK(shanghai)2022-0004]. The rodents were sedated using a 2% solution of sodium pentobarbital at a dosage of 110 mg per kilogram. After shaving and disinfecting the abdomen with hair removal cream and povidone-iodine, a 1.0-2.0 cm incision along midline under diaphragm was made to expose cecum. The process of CLP was used to create sepsis, involving partial ligation of the cecum and two punctures with an 18-gauge needle, followed by gentle compression to expel a minor amount of cecal material. After repositioning the cecum back into the abdominal cavity, the incisions in the muscle and skin were sutured with 4-0 silk. The control group experienced the identical process, excluding cecal ligation and puncture. Following the operation, the mice were revived with a subcutaneous injection of 1 ml of phosphate-buffered saline heated to 37°C. Twenty-four hours post-CLP surgery, lung samples and peripheral blood were gathered for subsequent examination was collected from mice. In addition, we also gave mice airway inhalation of LPS (Solarbio, China) at 10 mg/kg to induce the development of ARDS.

For the mice undergoing drug intervention, the following drugs were administered: SB 216763 (Yeasen, Shanghai, China) at a dosage of 3 mg/kg, Withaferin A at 2 mg/kg, sodium dichloroacetate (Macklin, Shanghai, China) at 10 mg/kg, Epothilone B (Aladdin, Shanghai, China) at 1 mg/kg, and the CX3CR1 inhibitor AZD8797 (Bidepharm, China) at 3 mg/kg, as well as PTGDS protein from MedChemExpress (MCE, NJ, USA) at 0.5 mg/kg. All drugs were administered via tail vein injection, and after 3 hours, the mice were subjected to modeling. Following modeling, serum and lung tissue samples were collected to assess the therapeutic effects of the drugs on the mice.

### 2.18 Enzyme-linked immunosorbent assay, H&E staining and lung damage assessment

After mice were anesthetized, blood was drawn from the heart, coagulated at 25°C for 2 h, and centrifuged (2,000 × g, 4°C for 20 min). Serum is then collected. The blood levels of the pro-inflammatory cytokine IL-6 was measured using an ELISA kit (Thermo Fisher Scientific, Waltham, MA, USA) following the manufacturer’s protocol with purified serum. According to the instructions of the H&E staining kit, lung tissues were encased in paraffin, dewaxed, rehydrated, and then stained using hematoxylin and eosin. The stained sections were then mounted on slides and examined under the optical microscope. The degree of lung injury was assessed using a semiquantitative scoring method (40). Specifically, lung damage is defined by four key factors: fluid buildup, hemorrhage, neutrophil presence or aggregation in the alveolar space or vessel wall, and the thickening of the alveolar wall and/or formation of a hyaline membrane. The standards for assessing pathological findings were: 0 for no damage; 1 for minor and limited damage (less than 25%); 2 for moderate damage (25-50%); 3 for extensive or significant damage (50-75%); and 4 for severe and most significant damage (over 75%). Each item was rated from 0 to 4. The four scores were then summed to determine the lung injury score, which ranged from 0 to 16. Two independent researchers evaluated the lung injury score, and if their assessments differed, the average of their scores was taken.

### 2.19 Statistical analysis

The data were analyzed using R (version 4.3.3). The “limma” package identified differentially expressed genes. For continuous variables, independent t-tests and one-way ANOVA were applied to normally distributed data, while the Mann-Whitney U test and Kruskal-Wallis test were used for non-normally distributed data. Results were expressed as mean ± standard deviation for normal distributions, and median (interquartile range) for non-normal distributions. Categorical variables were analyzed using chi-square or Fisher’s exact tests. Pearson correlation assessed the relationship between hub genes and clinical features, and logistic regression explored the diagnostic significance of CX3CR1, PTGDS, and PID1 in sepsis and ARDS. A multivariable Cox regression model was used to predict 28-day mortality in sepsis patients based on hub genes. Kaplan-Meier survival curves and log-rank tests compared overall survival rates. ROC curves (pROC package) were used for binary classification prediction, and Spearman correlation assessed the relationship between hub gene expression and immune cells. Experimental data were expressed as mean ± standard deviation from at least three independent experiments, and statistical significance was determined using unpaired two-tailed Student’s t-tests (*P < 0.05; **P < 0.01; ***P < 0.001). Statistical analysis and graphics were generated with GraphPad Prism 9.0, and figures were edited using Adobe Illustrator.

## 3 Results

### 3.1 The study framework and workflow

The workflow of the study design is depicted in **Figure 1**. First, we obtained sepsis and ARDS RNA-seq datasets from the GEO database (**Table 1**). Furthermore, we utilized comprehensive bioinformatics and WGCNA to identify gene modules, and emphasized crucial genes through three machine learning techniques: LASSO regression, Boruta and Random Forest. Then we found three critical hub genes (CX3CR1, PTGDS and PID1) and verified their diagnostic efficacy. Analysis of immune infiltration was then performed to figure out the relationship of three biomarkers and immune cells. We also constructed ceRNA network, PPI network and predicted TFs-hub genes interaction. Afterwards, we made predictions about the prospective medications or chemical compounds that may interact with hub genes. Additionally, we conducted molecular docking to confirm the interaction between the compounds and hub genes. We performed additional verification of the expression levels of crucial genes found in the scRNA-seq datasets for sepsis and ARDS. In order to confirm our bioinformatics results by RT-qPCR, we finally gathered our own PBMCs samples from healthy persons, sepsis patients, ARDS patients, as well as mice models. In addition, we also conducted different types of drug intervention on model mice and tested the therapeutic effects. It is anticipated that the association of genes identified here between sepsis and ARDS will offer novel insights into the biological mechanisms that underlie both diseases.

### 3.2 Identification of common DEGs between sepsis and ARDS

**Figure 2:**
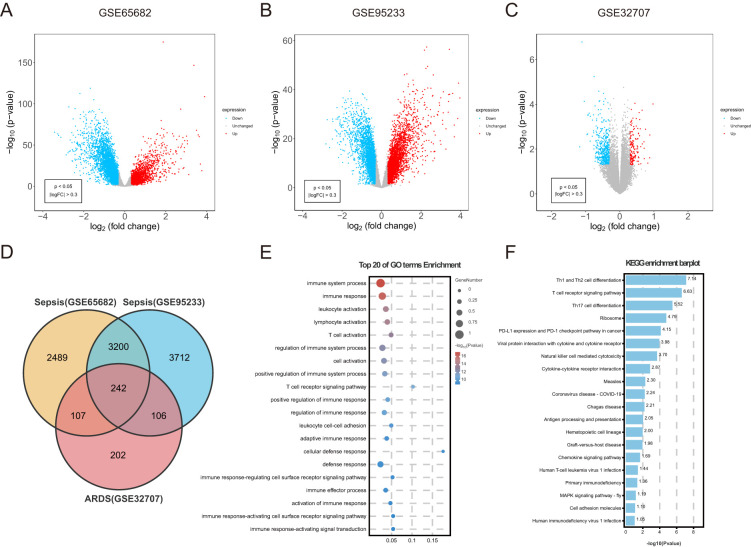
Identification of common DEGs between sepsis and ARDS, followed by GO and KEGG analysis. **(A)** DEGs shown on a volcano plot in GSE65682. **(B)** DEGs shown on a volcano plot in GSE95233. **(C)** DEGs shown on a volcano plot in GSE32707. **(D)** The shared DEGs between sepsis and ARDS by overlapping the DEGs of them. **(E)** GO enrichment analysis bubble graph for down-regulated DEGs. The larger bubble represents enrichment of more genes, and the increasing depth of red color represents more significant differences. **(F)** Histogram for KEGG enrichment in down-regulated DEGs.

An overall number of 6038 DEGs were discovered in the sepsis dataset GSE65682. Of these DEGs, 3621 were up-regulated, whereas 3641 were down-regulated. The sepsis dataset GSE95233 included 7260 DEGs, with 3620 indicating up-regulation and 3640 showing down-regulation. Furthermore, 657 DEGs related with ARDS were discovered, including 153 upregulated and 504 downregulated genes. Using the volcanic maps, the expression pattern of each DEG in connection to sepsis (**Figures 2A, B**) and ARDS (**Figure 2C**) was demonstrated. Venn diagrams displayed that 242 overlapping differentially expressed genes were identified from GSE65682 dataset, GSE95233 dataset and GSE32707 dataset (**Figure 2D**). Results of differential expression analysis suggested that sepsis and ARDS may share some mechanistic similarities.

### 3.3 Examination of common genes and their functional significance

To understand the biological functions and pathways linked to sepsis and ARDS, GO enrichment and KEGG pathway analyses were conducted on the 242 common differentially expressed genes. During the GO enrichment analysis, the down-regulated DEGs were mainly linked to immune system activities, leukocyte activation, and T cell activation (**Figure 2E**). This implies that immune system suppression and the programmed death of immune cells might be crucial in the simultaneous onset of sepsis and ARDS. For these up-regulated genes, DEGs mainly contributed to the proteasome-mediated ubiquitin-dependent protein degradation and protein breakdown processes.

Additionally, KEGG pathway analysis demonstrated that these upregulated genes had a significant connection with platelet activation, autophagy, and human T-cell leukemia virus infection (**Supplementary Figure S1B**). Apoptosis caused by sepsis occurs through pathways involving death receptors and mitochondria. Additionally, KEGG analysis indicated that these frequently down-regulated genes were involved in Th1 and Th2 cell differentiation, NK cell-mediated cytotoxicity, antigen processing and presentation, and cytokine-cytokine receptor interactions (**Figure 2F**). Immunosuppression is increasingly acknowledged as a significant contributor to mortality in sepsis (41). Likewise, patients with ARDS often experience immunosuppression, and their mortality rates are higher compared to immunocompetent individuals (42).

### 3.4 Identifying crucial modules using WGCNA

**Figure 3:**
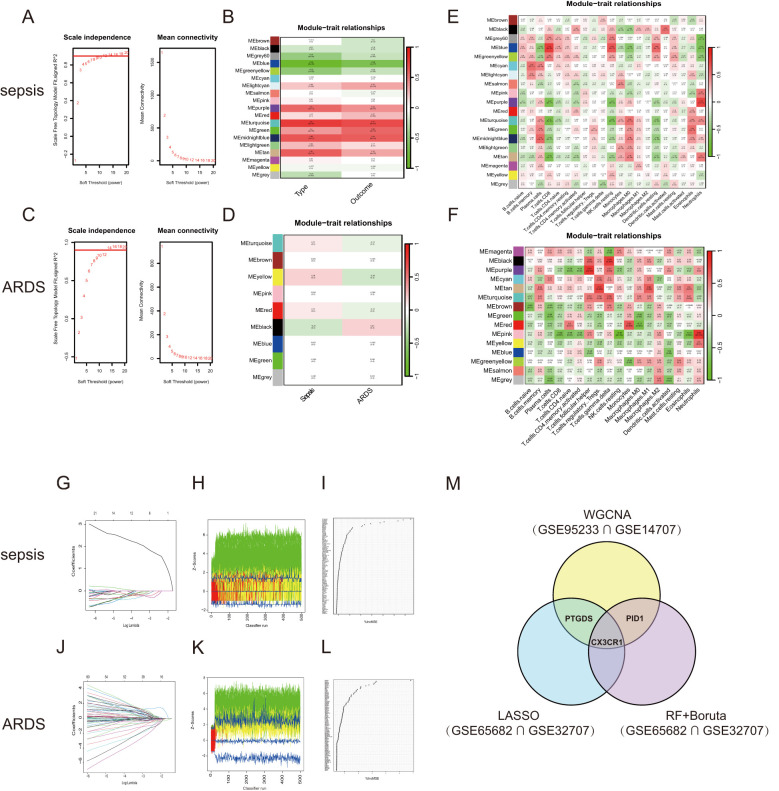
WGCNA and Machine learning in screening candidate diagnostic biomarkers for sepsis and ARDS. **(A)** Identifying the soft-threshold power in sepsis using GSE95233. **(B)** Interconnections among modules and traits in sepsis. Correlation coefficients and P values are incorporated in each cell. **(C)** Estimation of the soft-threshold power for ARDS using GSE10474. **(D)** Associations between modules and traits in ARDS. **(E)** Correlation heatmap between module eigen genes and immune infiltration cells in sepsis. **(F)** Correlation heatmap between module eigen genes and immune infiltration cells in ARDS. **(G, J)** The LASSO regression was used to identify the minimum and lambda values for diagnostic biomarkers for sepsis and ARDS. **(H, K)** The utilization of the Boruta aimed at identifying common diagnostic genes of sepsis and ARDS. **(I, L)** Genes in sepsis and ARDS identified for their discriminatory power in the RF algorithm. **(M)** Venn diagram shows that three candidate diagnostic genes are identified via the above three algorithms and WGCNA.

WGCNA was conducted to explore the possible correlations among significant genes and illnesses. Using the soft-thresholding technique, a co-expression network was constructed, with the parameter β playing a crucial role in maintaining a scale-free topology (**Figures 3A, C**). Gene expression data typically produce biological networks that exhibit a scale-free topology. In the sepsis group, a fit index exceeding 0.9 indicated a scale-free topology, with the parameter β set to 7. Utilizing the adjacency function, an adjacency matrix was generated, followed by hierarchical clustering based on the TOM dissimilarity measure (**Supplementary Figures S1C, E**).

We looked for the most significant connections after correlating sepsis clinical features to modules. **Figure 3B** showed the correlation values between the modules and traits (type: Sample types of healthy or sepsis patient, and outcome: whether sepsis patients died). This study found that the turquoise module had the most significant link to the onset of sepsis and its unfavorable outcome. We further selected 1478 genes with |MM| > 0.6 and |GS| > 0.6. Following the examination of gene module interactions, topological overlap matrix charts for a gene network were created using the related hierarchical cluster dendrograms and modules (**Supplementary Figure S1D**). **Figure 3E** illustrated the relationship between gene modules and immune cell characteristics in sepsis. Likewise, the ARDS cohort was subjected to WGCNA, pinpointing β = 14 as the optimal soft power value (**Figure 3C**). The dark module, containing 3351 genes, showed the strongest positive association with ARDS occurrence (**Figure 3D**). **Figure 3F** demonstrated the connections between various gene modules and multiple immune cell characteristics in ARDS. The genes found in the crucial modules of both groups could act as potential markers specific to certain cell types.

### 3.5 Identification of shared diagnostic markers using machine learning techniques

We applied three machine learning techniques—LASSO regression, Random Forest, and Boruta—to the GSE65682 and GSE32707 datasets to pinpoint the top predictors for sepsis and ARDS. We utilized the ‘glmnet’ library to perform LASSO regression analysis (**Figures 3G, J**; **Supplementary Figures S1G, H**). To enhance the identification of diagnostic biomarkers, we developed a random forest (RF) model using the ‘randomForest’ package in R, as shown in **Figures 3I, L**. Additionally, we employed the Boruta algorithm for further analysis (**Figures 3H, K**; **Supplementary Figures S1I, J**). Combining the findings from both algorithms and WGCNA, three shared biomarkers (CX3CR1, PID1 and PTGDS) were discovered for both the sepsis and ARDS groups. A Venn diagram (**Figure 3M**) was used to display the overlap of the top three critical genes identified by LASSO regression, Random Forest, Boruta, and WGCNA. We proposed that these three genes could be linked to the onset of sepsis and ARDS, sharing a mutual connection.

### 3.6 Creating a predictive tool for diagnosing sepsis and ARDS, along with assessing its accuracy

**Figure 4:**
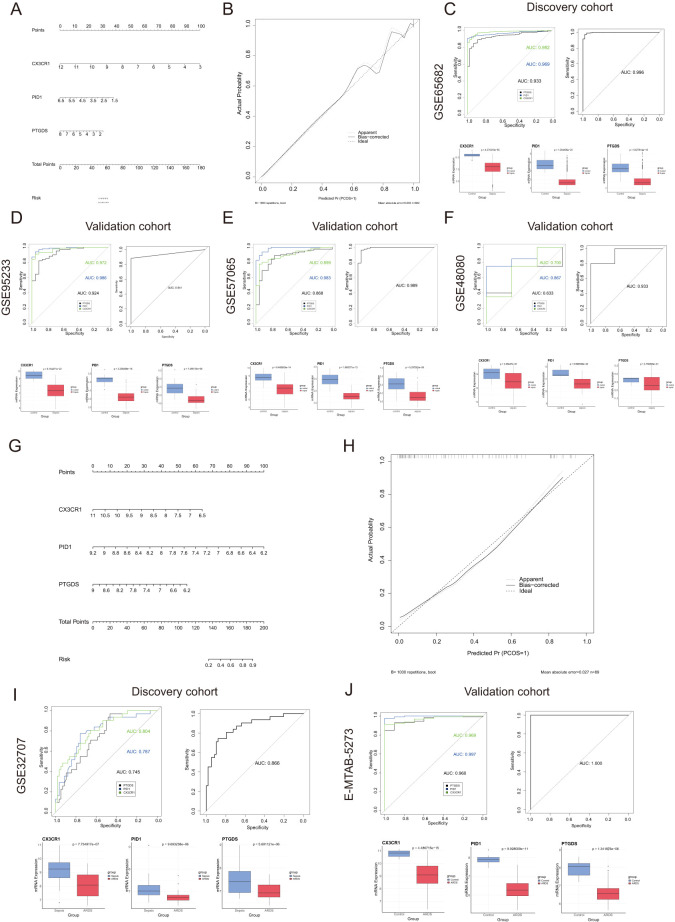
Nomogram construction and the diagnostic value evaluation in sepsis and ARDS. **(A)** Nomogram constructed based on the diagnostic biomarkers for risk prediction of sepsis using GSE65682. **(B)** Calibration curve for assessing the performance of the nomogram. **(C–F)** ROC curve and the CX3CR1, PID1 and PTGDS expression levels in the discovery cohort and the validation cohort in sepsis. **(G)** The visible nomogram for diagnosing ARDS using GSE32707. **(H)** Calibration curves were used to evaluate the predictive performance of the nomogram model. **(I, J)** The ROC curve of each candidate gene and the gene expression levels show the significant ARDS diagnostic value.

A predictive model for sepsis was created using three crucial genes—CX3CR1, PID1, and PTGDS—to enhance diagnostic precision. Each risk factor received a score, and these individual scores were aggregated to determine the total score for estimating the probability of sepsis in each patient (**Figure 4A**). **Figure 4B** illustrates that the calibration curves in the dataset closely matched the standard curve, demonstrating the nomogram’s high precision in forecasting sepsis. The ROC curve analysis indicated an AUC of 0.966 for the risk score within the discovery dataset, as shown in **Figure 4C**. Similarly, the risk score’s AUC was 0.941, 0.989, and 0.933 when the ROC curve was generated with the validation dataset (**Figures 4D–F**). **Figures 4C–F** depict the expression levels of the three critical genes. The results suggested that the risk-score model has strong predictive capabilities, with the three main diagnostic biomarkers being crucial in the progression of sepsis.

Similarly, we developed a nomogram specifically for ARDS patients utilizing these three unique genes (**Figure 4G**). The model achieved a remarkable AUC score of 0.866 when analyzing the ROC curve (**Figure 4I**). Additionally, we confirmed the expression of the critical genes in an external dataset (E-MTAB-5273, **Figure 4J**). **Figure 4H** illustrated that the calibration curve results validated the remarkable precision of the nomogram model in predicting patient outcomes for ARDS. As shown in **Figures 4I**, **J**, the levels of CX3CR1, PID1 and PTGDS gene expression were found to be lower in the ARDS group induced by sepsis compared to both the sepsis-only and control groups.

### 3.7 Creation and validation of a forecasting model for outcomes in sepsis and ARDS

**Figure 5:**
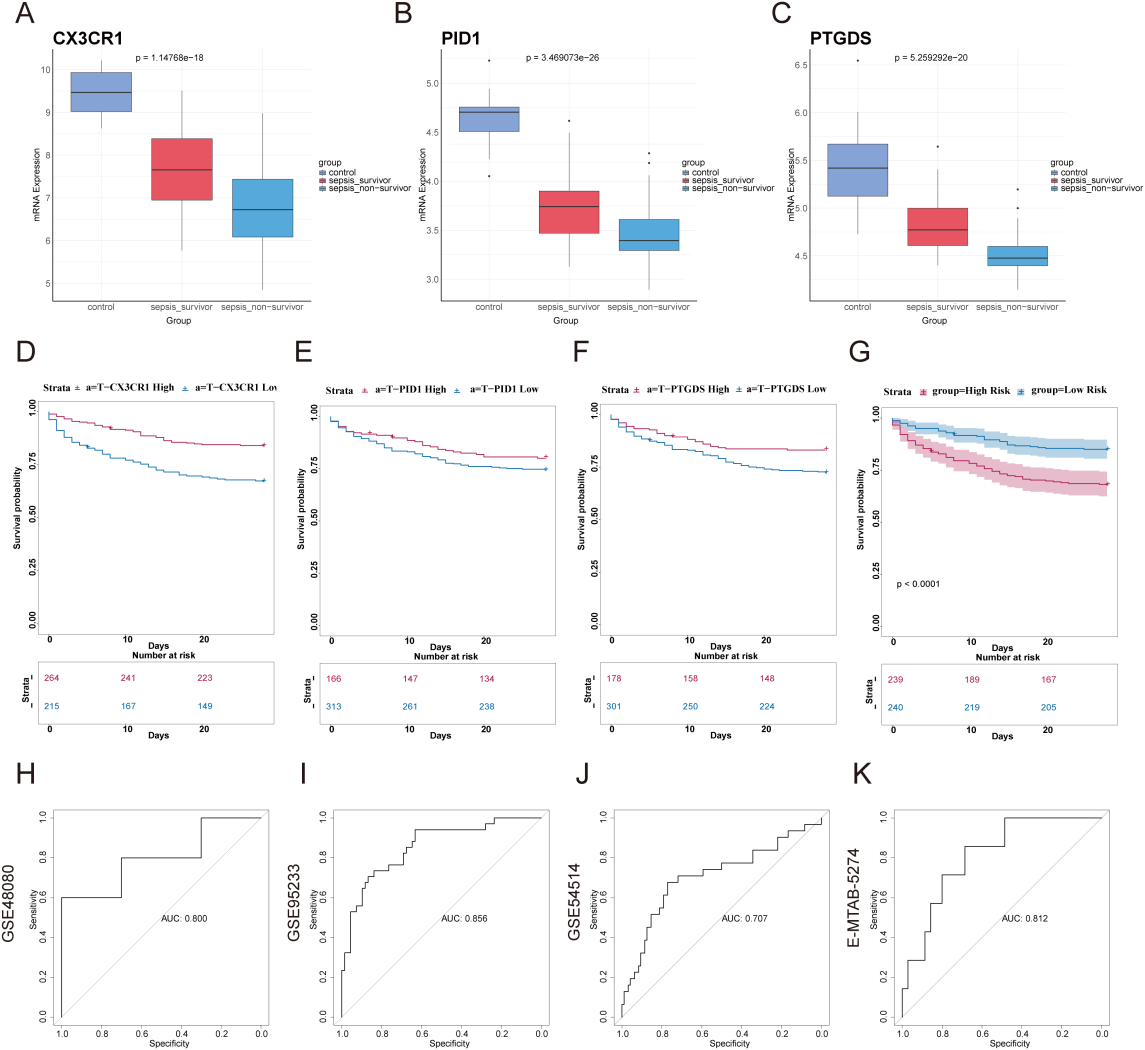
Construction of prognostic model based on hub genes and prognostic efficacy in sepsis and ARDS. **(A–C)** The expression levels of the three genes were analyzed in the healthy control group, sepsis survivor group, and sepsis non-survivor group from the dataset GSE95233. **(D–F)** Using GSE65682, Kaplan–Meier survival analysis was performed to assess the prognostic value of these candidate genes in sepsis. **(G)** Kaplan-Meier survival analysis of 28-day mortality between high-risk (red) and low-risk (blue) groups in sepsis. The color of each survival line indicated the 95% Cl of probability of survival at each time point. **(H)** The ROC curve showed the AUC value of the prognostic model in the GSE48080 sepsis dataset. **(I)** The ROC curve for the GSE95233 sepsis dataset. **(J)** The ROC curve for the GSE54514 sepsis dataset. **(K)** The ROC curve for the E-MTAB-5274 ARDS dataset.

We categorized the sepsis dataset by patient survival outcomes and examined the mRNA expression levels of the genes CX3CR1, PID1, and PTGDS. Among the three genes, the highest expression levels were observed in the healthy control group, with the sepsis survivor group showing intermediate levels, and the sepsis non-survivor group exhibiting the lowest levels (**Figures 5A–C**). This implied that these genes are linked to the prognosis of sepsis patients. Kaplan-Meier curves indicated that low levels of CX3CR1, PID1, and PTGDS were correlated with poor overall survival (OS) (**Figures 5D–F**). A multivariate Cox regression analysis incorporated these genes to develop a prognostic model aimed at forecasting 28-day mortality in individuals with sepsis. The formula is presented in **Additional file 1: Table S1**. According to the median risk score, 479 sepsis patients were divided into high-risk (n=239) and low-risk (n=240) categories (**Supplementary Material: Table S1**). According to **Figure 5G**, the 28-day mortality rate was significantly elevated in the high-risk cohort. The ROC curve showed an AUC of 0.800, indicating good model accuracy for predicting 28-day mortality (**Figure 5H**). External validation using the GSE95233 and GSE54514 datasets showed AUC values of 0.856 and 0.707, respectively (**Figures 5I, J**). Moreover, the model reliably predicted ARDS outcomes, achieving AUC scores of 0.812 (**Figure 5K**). Consequently, this 3-gene signature may effectively predict the prognosis of sepsis and ARDS, further supporting its clinical value.

We also analyzed the expression of these three genes in correlation with key clinical outcomes (death outcome, SOFA score at 24 hours after admission, number of organ failures, etc.) in different datasets. We found a negative correlation between the expression of the three genes, CX3CR1, PID1 and PTGDS, and various adverse clinical outcomes (**Supplementary Material: Table S2**). In the GSE95233 dataset, gene expression was negatively correlated with mortality (correlation coefficients: -0.450, -0.455, -0.554, p < 0.01). In GSE185263, gene expression was also negatively correlated with SOFA score (correlation coefficients: -0.261, -0.239, -0.149, p < 0.01). In GSE63311, CX3CR1 and PTGDS expression were negatively correlated with organ failure counts (correlation coefficients: -0.476, -0.324, p < 0.01), while PID1 was not significant (p > 0.05). In addition, we found that the expression of the three genes was also correlated with sepsis survival, SOFA score, clinical diagnosis of sepsis, and number of organ failures, and that their expression levels tended to decrease with worsening prognosis, further supporting their value as potential biomarkers(**Supplementary Figures S2A–F**).

### 3.8 Investigating the infiltration of immune cells and its relationship to common hub genes

**Figure 6:**
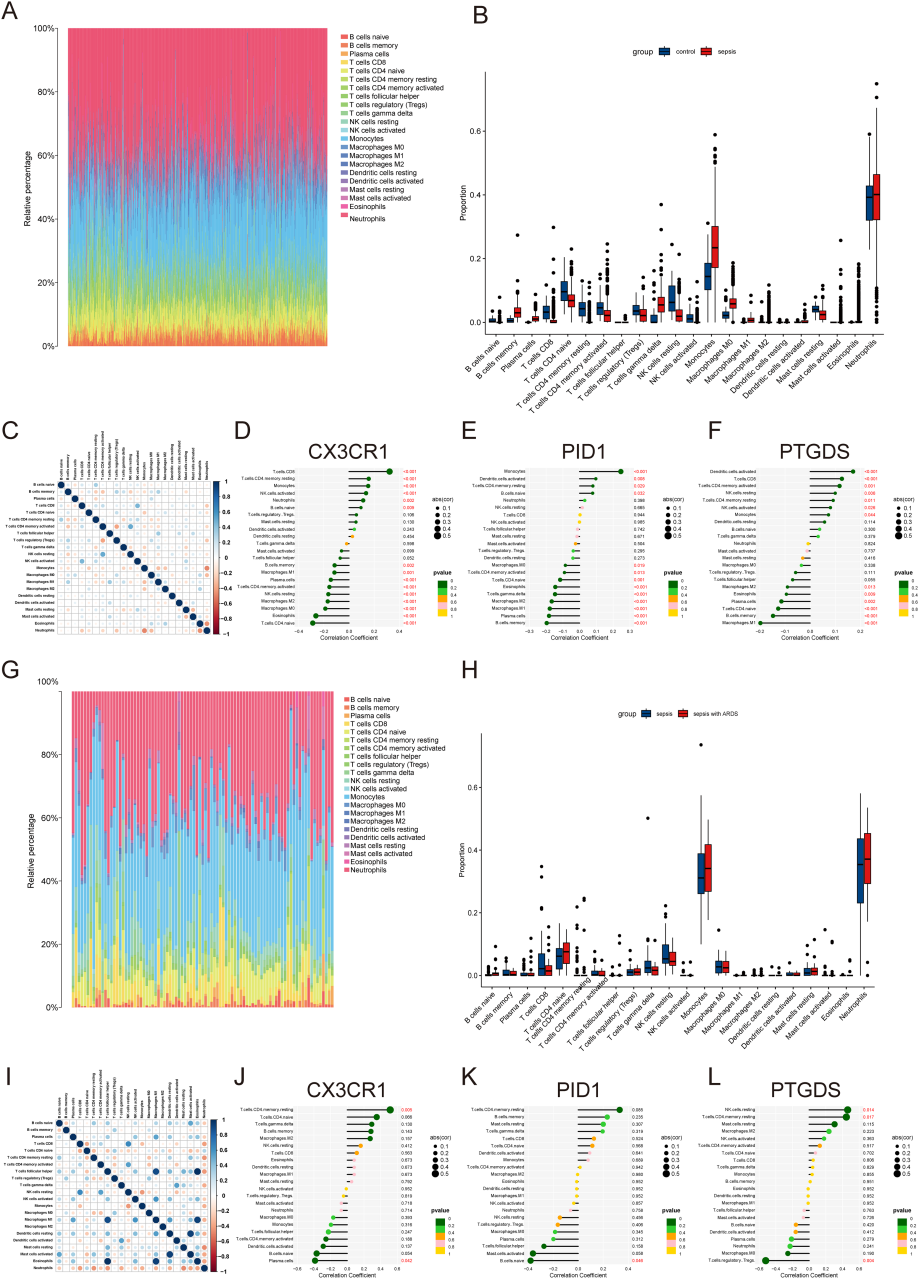
Immune infiltration landscape in sepsis and ARDS. **(A)** Proportional graph of 22 immune cells in GSE65682. **(B)** The proportion of 22 kinds of immune cells in different samples visualized from the barplot. **(C)** Correlation of 22 immune cells by compositions. Both horizontal and vertical axes demonstrate immune cell subtypes. **(D–F)** Correlation of CX3CR1, PID1 and PTGDS with infiltrating immune cells in sepsis group. **(G)** Proportional graph of 22 immune cells in GSE32707. **(H)** Barplot indicating the comparison of 22 types of immune cells between the sepsis and sepsis with ARDS groups. **(I)** Heatmap of correlations among 22 immune cells. **(J–L)** The association between CX3CR1, PID1 and PTGDS expression with different immune cell infiltration in the ARDS group.

The preliminary enrichment study indicated a connection between the core genes CX3CR1, PID1, and PTGDS and processes related to inflammation and immunity. As a result, a comprehensive examination of immune cell information in different groups was carried out using CIBERSORT. In instances of sepsis, there was an elevated count of monocytes, macrophages, memory B cells, and neutrophils, whereas the healthy control group exhibited a higher number of CD8 T cells, naive CD4 T cells, and resting NK cells (**Figures 6A, B**). The correlation study revealed positive associations between activated CD4 memory T cells and resting NK cells, along with connections between CD8 T cells and activated NK cells. Conversely, a negative correlation was observed between monocytes and resting neutrophils (**Figure 6C**).

Further investigation revealed correlations across the hub genes to specific immune cell categories. CX3CR1 was found to have a positive association with activated CD8 T cells and a negative one with naive CD4 T cells (**Figure 6D**). PID1 showed a positive relationship with monocytes and a negative one with memory B cells (**Figure 6E**). PTGDS exhibited positive correlations with activated dendritic cells and negative correlations with M1 macrophages (**Figure 6F**). In ARDS, we analyzed 22 types of immune cells in datasets of sepsis and sepsis with ARDS (**Figures 6G, H**). Patients suffering from ARDS and sepsis had elevated levels of monocytes, neutrophils, and naive CD4 T cells, while those with only sepsis had higher counts of CD8+ T cells and resting NK cells. Additionally, CX3CR1 and PID1 levels were positively linked to resting CD4 memory and naïve CD4 T cells, but inversely related to plasma cells and naïve B cells (**Figures 6I–K**). PTGDS showed a positive association with activated resting NK cells and mast cells, while it was inversely related to Tregs (**Figure 6L**).

### 3.9 The GSEA of hub genes

**Figure 7:**
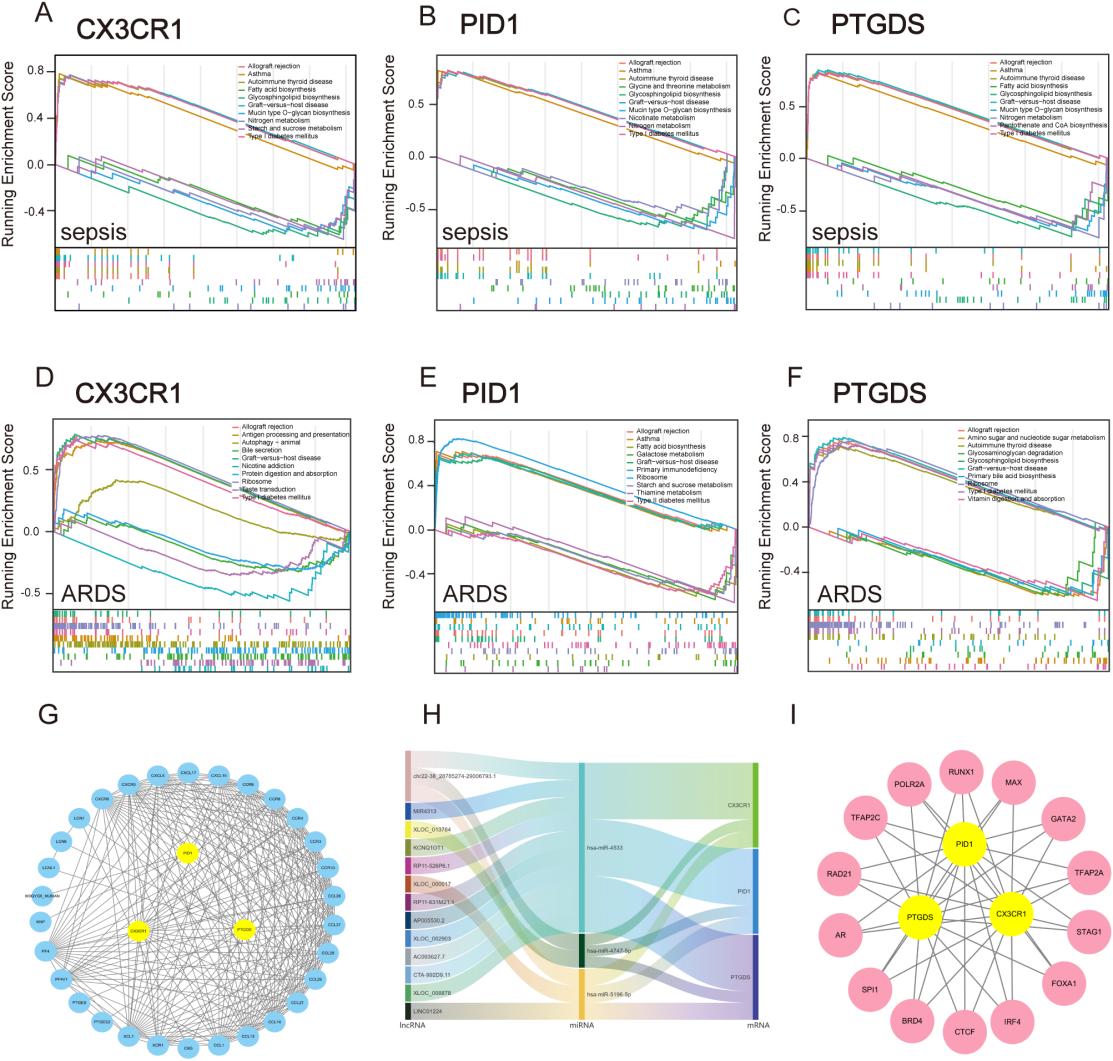
GSEA analysis and construction of TFs, ceRNA and PPI network based on three marker genes. **(A–C)** GSEA analysis for CX3CR1, PID1 and PTGDS in the sepsis group in GSE65682. **(D–F)** GSEA analysis for CX3CR1, PID1 and PTGDS in ARDS group in GSE32707. **(G)** STRING-based PPI network of three hub genes. **(H)** The Sankey diagram displays the lncRNA–miRNA–mRNA regulatory module, with lncRNAs on the left, miRNAs in the middle, and mRNAs on the right. **(I)** Network of shared TFs based on three hub genes.

Subsequently, we employed GSEA analysis of the three biomarkers in sepsis and ARDS datasets, respectively. We discovered notable associations between the central genes and multiple signaling pathways. For sepsis (**Figures 7A–C**), CX3CR1, PID1 and PTGDS are strongly associated with immune pathways such as “Allograft rejection” and “Asthma”. They are also involved in metabolic pathways like “Glycosphingolipid biosynthesis,” “Nitrogen metabolism,” and “Type I diabetes mellitus”. Interestingly, CX3CR1 and PTGDS are involved in “Fatty acid biosynthesis”, whereas PID1 specifically affects “Glycine, serine and threonine metabolism” and “Nicotinate and nicotinamide metabolism”. Additionally, PTGDS is specifically enriched in “Pantothenate and CoA biosynthesis”. For ARDS (**Figures 7D–F**), these genes similarly highlight their involvement in immune response and metabolic pathways.

These findings suggest that CX3CR1, PID1 and PTGDS are integral to both immune regulation and metabolic processes, playing multifaceted roles in conditions like sepsis and ARDS. Their participation in various immune mechanisms underscores their promise as indicators or treatment targets for diseases related to the immune system.

### 3.10 Analysis of transcription factors, ceRNA regulatory networks and protein-protein interactions for three key genes

Significant transcriptional changes and key regulatory molecules associated with central genes were discovered through an online approach. A trio of core genes was submitted to STRING to construct the protein interaction network, and the subsequent data was transferred to Cytoscape for visual depiction. The MCODE plugin was employed to detect significant clusters, revealing a notable module with 35 nodes and 245 connections, as shown in **Figure 7G**. By integrating data from the miRDB, miRabel, miRWalk, and TargetScan databases for the three central genes, we discovered three common unique miRNAs: hsa-miR-4533, hsa-miR-5196-5p, and hsa-miR-4747-5p. Furthermore, the lncBase database identified 15 lncRNAs involved in regulating all miRNAs. Ultimately, a ceRNA network was established, comprising 15 lncRNAs, 3 miRNAs, and 3 crucial genes (**Figure 7H**). Through the Cistrome Data Browser database, we obtained Top 20 transcription factors of CX3CR1, PID1 and PTGDS, respectively. Subsequently, we obtained shared TFs and hub genes regulatory network using the MCODE plugin (**Figure 7I**). These findings enable deeper exploration into the distinct roles and functions of transcription factors, regulated RNAs, and interacting proteins within the framework of hub genes. This, in turn, enhances our comprehension of biological mechanisms and potential therapeutic targets in the diseases under investigation.

### 3.11 Discovery of potential small-molecule drugs and molecular docking for treating sepsis and ARDS

**Figure 8:**
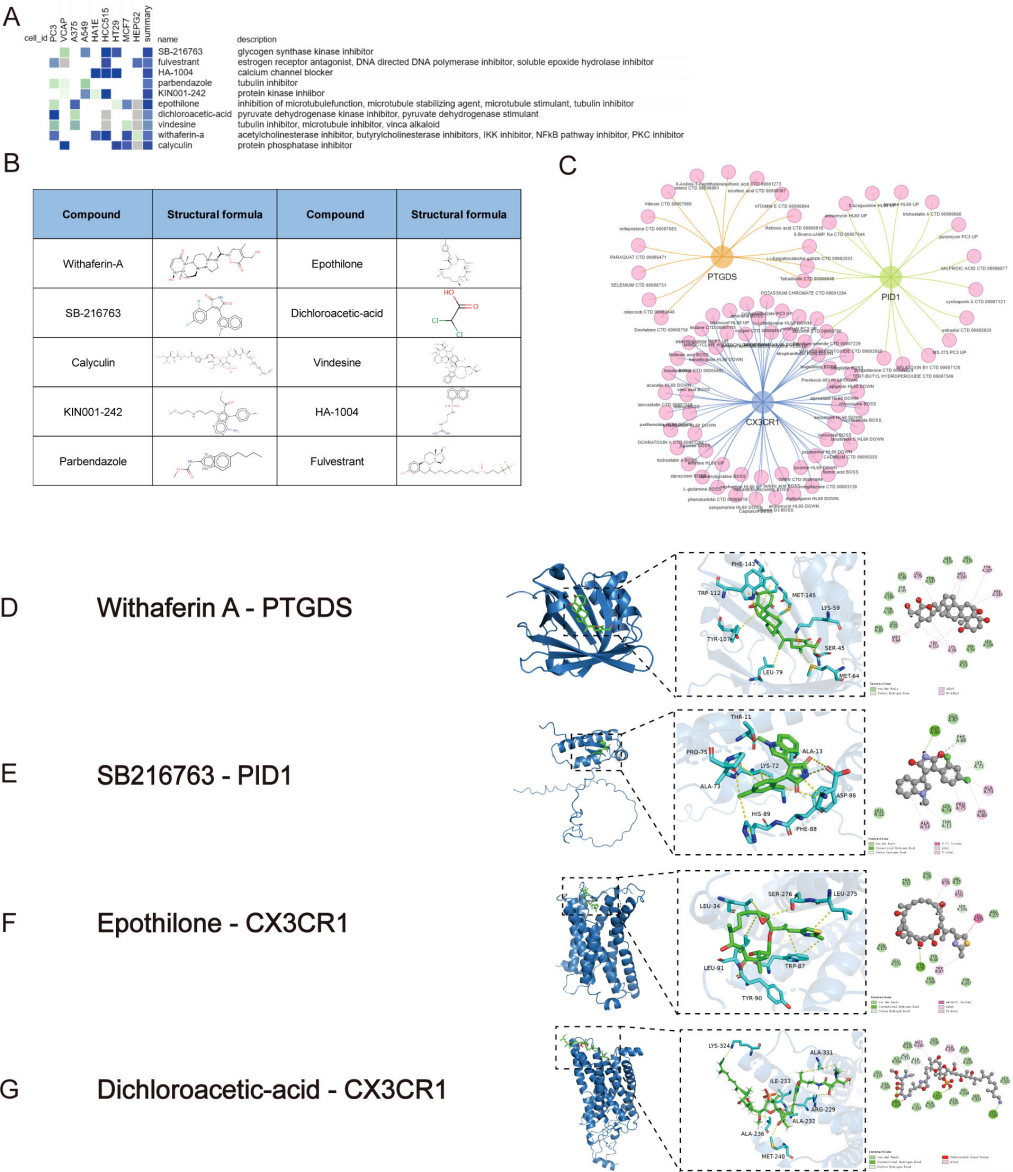
Screening of promising chemicals for the treatment of sepsis and ARDS using CMap and Enrichr. **(A)** The heatmap presenting the top10 compounds with the most significantly negative enrichment scores in 10 cell lines based on CMap analysis. **(B)** The chemical structures of those 10 compounds were shown in CMap database. **(C)** Potential drugs targeting shared DEGs in Enrichr database. **(D)** The docking result of Withaferin A with PTGDS. **(E)** The docking result of SB216763 with PID1. **(F)** The docking results of hub genes encoded proteins with small molecular compounds. The docking result of Epothilone with CX3CR1. **(G)** The docking result of Dichloroacetic-acid with CX3CR1.

To explore possible small-molecule drugs that could have therapeutic benefits for sepsis and ARDS patients, DEGs were analyzed using the CMap database to identify potential medicines that might counteract the changes in pathogenic gene expression. After an extensive investigation, the ten leading compounds—such as withaferin-a, epothilone, SB-216763, parbendazole, KIN001-242, fulvestrant, dichloroacetic acid, vindesine, HA-1004, and calyculin—were identified as promising pharmacological candidates for treating sepsis and ARDS due to their high negative scores. **Figures 8A, B** illustrated the pathways and chemical structures of the ten compounds. A screening method using Enrichr was also implemented to discover possible drugs that target key genes. Promising compounds were identified using transcriptional data obtained from DSigDB. Comprehensive results of all drug candidates can be found in **Figure 8C**.

The MOE 2019 program was used to perform molecular docking analyses, with the objective of investigating the interactions between different drugs and their respective targets. According to the docking results, Withaferin A, SB216763, Epothilone and Dichloroacetic Acid can bind to the binding domains of PTGDS, PID1, and CX3CR1 target proteins, respectively. The docking energies are -6.9451, -5.6481, -6.9810, and -3.9131 kcal/mol, respectively (**Figures 8D–G**). Docking energy values below -4.25 kcal/mol suggest some binding activity, values under -5.0 kcal/mol indicate significant binding, and values below -7.0 kcal/mol indicate strong binding. The docking results show that Withaferin A-PTGDS, Epothilone-CX3CR1 and SB216763-PID1 have good binding activity. These chemical compounds possess significant potential to inhibit or even prevent the progression and development of sepsis and ARDS.

### 3.12 Single cell analysis of sepsis and ARDS

**Figure 9:**
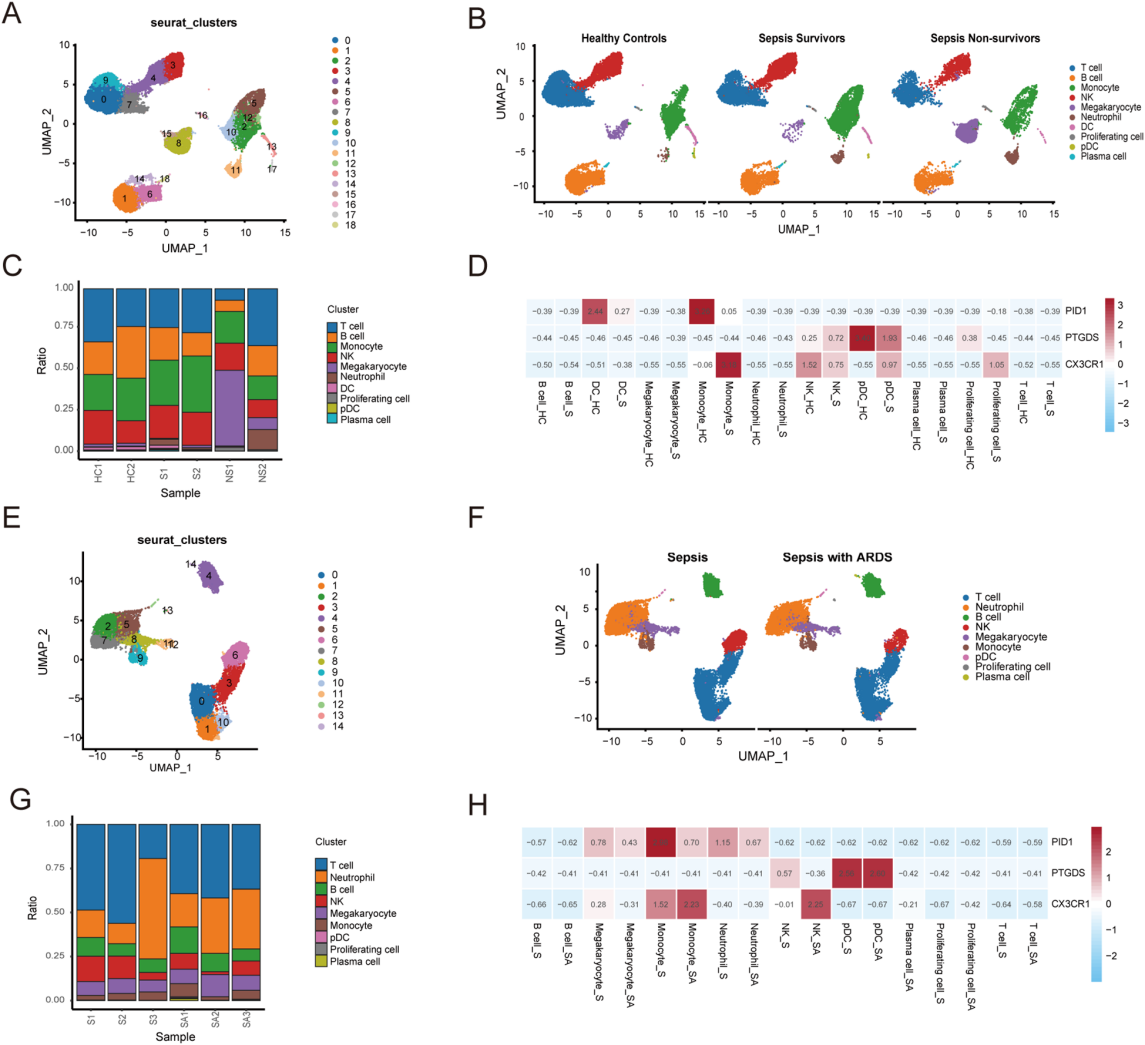
Subsets within cell types identified through single-cell RNA sequencing of sepsis and ARDS. **(A)** UMAP plot of 18 cell clusters grouped using the Seurat package in sepsis group of the GSE167363 dataset. **(B)** The UMAP plot illustrates the distribution of cell clusters among the healthy control, sepsis survivor, and sepsis non-survivor group samples. **(C)** Distribution of cell types between healthy control and groups displayed in the cumulative histogram. **(D)** Expression of marker genes in each cell type. **(E)** UMAP plot of 14 cell clusters grouped using the Seurat package in ARDS group of the GSE151263 dataset. **(F)** The UMAP plot illustrates the distribution of cell clusters among the only-sepsis and sepsis with ARDS group samples. **(G)** Distribution of cell types between sepsis and ARDS groups displayed in the cumulative histogram. **(H)** Expression of marker genes in each cell type.

A single-cell analysis of the GSE167363 dataset was conducted to further investigate sepsis. This research included a total of six samples from the healthy control, survivor, and non-survivor categories. The distribution of cells across the groups was fairly consistent, suggesting no significant batch effects were present among the samples. We categorized all cells into 18 distinct clusters based on their gene expression profiles (**Figure 9A**). Using these marker genes, we identified ten major cell types: T cells, B cells, monocytes, NK cells, megakaryocytes, neutrophils, dendritic cells (DCs), proliferating cells, plasmacytoid dendritic cells (pDCs), and plasma cell (**Figure 9B**). **Figure 9C** depicted the distribution of these cell types across the clusters. The results further confirmed that CX3CR1, PID1 and PTGDS were significantly highly expressed in Monocytes, DCs, pDCs and NK (**Figure 9F**). We found that the survivor and healthy control groups had a significantly higher proportion of NK cells, T cells and monocytes compared to the non-survivor group. In contrast, the non-survivor group showed a markedly higher proportion of neutrophils and megakaryocytes (**Figure 9D**).

Simultaneously, we conducted a similar analysis on the singlecell dataset GSE151263 for patients with sepsis alone and those with sepsis combined with ARDS (**Figures 9E**, **G**, **H**). Notably, as illustrated in the **Figure 9F**, patients with sepsis combined with ARDS exhibited significantly fewer NK cells and T cells compared to those with sepsis alone. This observation highlights a potential disparity in immune cell distribution between the two patient groups. The reduced counts of NK cells and T cells in patients suffering from sepsis and ARDS suggest that these immune elements may have a pivotal function in regulating the advancement of diseases and impacting the outcomes of patients.

### 3.13 RT-qPCR verification of human and mice samples, inflammation and lung injury in mice after drug administration

**Figure 10:**
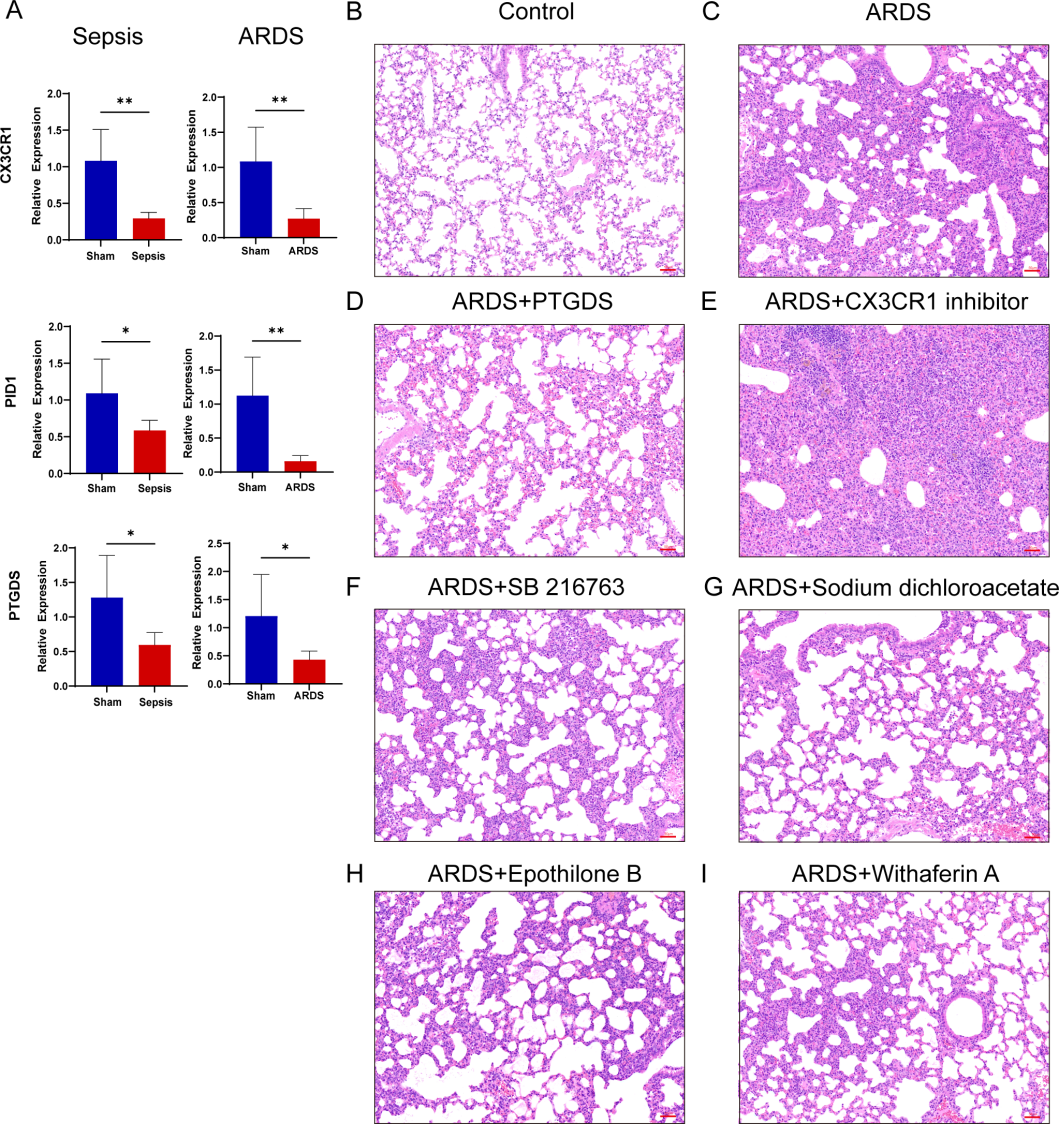
RT-qPCR verification of mice samples, inflammation and lung injury in mice after drug administration. **(A)** Transcriptional expression in PBMCs from sepsis (n=6), sepsis-induced ARDS (n=6) mice and sham groups (n=6) of CX3CR1, PID1 and PTGDS relative to GAPDH. Data are presented as the mean ± standard deviation. *P < 0.05, **P < 0.01. **(B–I)** HE staining results of lung tissue of mice in control group, LPS-induced lung injury group and after treatment with various drugs (Images were magnified at 200×, Scale bar = 50 μm).

**Figure 11:**
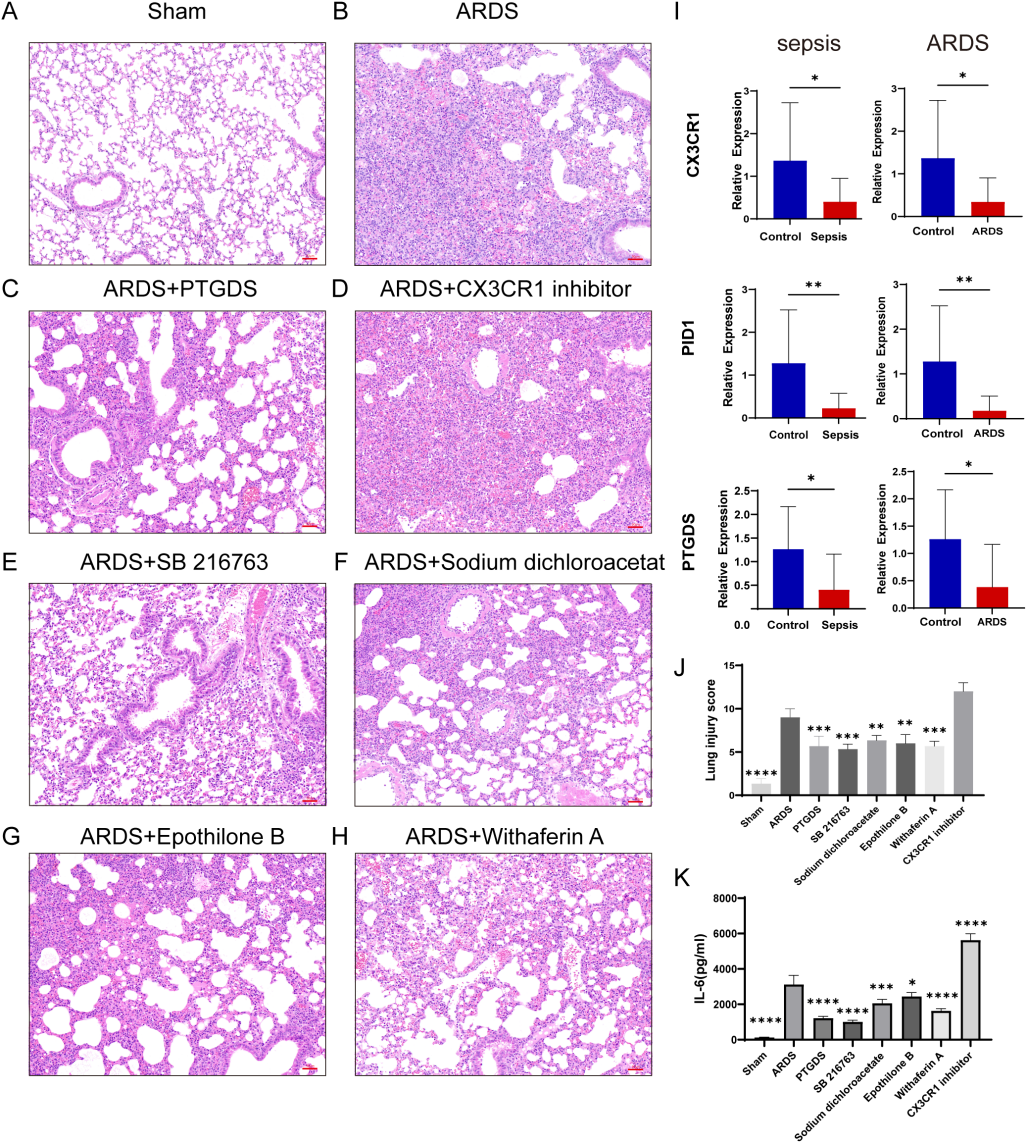
RT-qPCR verification of human samples, inflammation and lung injury in mice after drug administration. **(A–H)** HE staining results of lung tissue in sham group, CLP-induced lung injury group and postoperative treatment with various drugs (Images were magnified at 200×, Scale bar = 50 μm). **(I)** Transcriptional expression in PBMCs from sepsis (n=16) and ARDS (n=15) patients and healthy controls (n=12) of CX3CR1, PID1 and PTGDS relative to GAPDH. The P-value was obtained by comparing the two groups. **(J)** Lung injury score of HE staining in different groups (n=3). **(K)** Serum IL-6 levels in different groups of mice were detected by ELISA (n=3). Data are presented as the mean ± standard deviation. *P < 0.05, **P < 0.01, ***P < 0.001, ****P < 0.0001. The P-value was compared with the sham operation group.

In order to validate our results, we induced sepsis in mice using the CLP model, and some were displaying acute lung injury. Lung tissues were then collected for HE staining. In the sham group, the lung tissue of mice showed a normal alveolar architecture, featuring consistent alveolar lumen size, thin septa, absence of edema or inflammatory cells, and evenly stained cytoplasm with distinct nuclei (**Figure 11A**). In contrast, the ARDS group of mice showed signs of alveolar congestion, inflammatory exudates, and thickened alveolar walls, resulting in a much higher lung injury score compared to the sham group (**Figures 11B, J**, p < 0.0001). In addition, we collected blood samples from both humans and mice. The RT-qPCR was performed on PBMCs from healthy controls, septic patients and ARDS patients. In line with the data analysis, our results showed that three key genes had less expression in the PBMCs of patients with sepsis and ARDS (**Figure 11I**). We also performed RT-qPCR on PBMCs samples from sepsis and sepsis-induced ARDS model mice, obtaining consistent results (**Figure 10A**). Consequently, delving deeper into their functions in sepsis and ARDS is crucial for the diagnosis and treatment of these conditions.

In addition, after treatment with four predictive drugs and PTGDS protein, the lung damage of mice was reduced to varying degrees, interstitial edema and inflammatory cell infiltration were significantly reduced, and systemic inflammation was also reduced (**Figures 11C, E–H**). We obtained similar results by allowing mice to induce ARDS by airway inhalation of LPS and administering different drugs (**Figures 10B–D, F–I**). However, after administration of CX3CR1 inhibitors to mice, pathological lung damage was aggravated, alveolar wall thickness increased, the number of neutrophils increased, lung tissue bleeding worsened, and serum IL-6 levels also increased (**Figures 10E**, **11D, J, K**).

## 4 Discussion

Sepsis is a severe global public health issue (43). It can induce ARDS, and the two conditions may be closely connected, though the specific mechanisms remain unclear (3, 8, 44). In this study, differential gene analysis, WGCNA, and machine learning were utilized to identify novel gene signatures related to sepsis and ARDS (CX3CR1, PID1, and PTGDS). Reduced levels of these three key genes might be linked to the diagnosis and unfavorable outcomes of sepsis and ARDS.

We successfully identified three hub genes: CX3CR1, PID1, and PTGDS. CX3CL1 (fractalkine) is a transmembrane protein and chemokine, and CX3CR1 is the only receptor for CX3CL1 expressed in leukocyte subsets. The CX3CR1-CX3CL1 signaling pathway mediates immune responses, inflammation, cell adhesion, and chemotaxis functions (45, 46). A forward-looking, multi-site, observational research identified CX3CR1 as the gene with the most significant differential expression between those who survived septic shock and those who did not. Lower levels of CX3CR1 mRNA were linked to higher death rates in ICU patients, potentially due to greater adhesion of inflammatory monocytes and a diminished inflammatory response (47, 48). Our survival analysis suggests that elevated expression of CX3CR1 is associated with better prognosis in sepsis and ARDS. Recent research indicates that administering CX3CL1 can modulate the inflammatory response in mice with sepsis (49). Our GSEA analysis demonstrates the association of CX3CR1 with autophagy regulation highlights its importance in controlling inflammation and enhancing immune responses, which is crucial for improving patient prognosis (50). This suggests that CX3CR1 could be a novel therapeutic target. The protein encoded by the PTGDS gene is a glutathione-independent prostaglandin D synthase that catalyzes the conversion of prostaglandin H2 (PGH2) to prostaglandin D2 (PGD2). PGD2 efficiently inhibits platelet aggregation and aids in mast cell maturation, thus being essential for vascular functions (51). Recent studies indicate that PTGDS possesses anti-inflammatory and pro-resolving characteristics, and its increased expression offers protection against pneumonia caused by Pseudomonas aeruginosa, suggesting that PTGDS is crucial in innate immune reactions (52). Moreover, GSEA analysis results show that PTGDS is implicated in the regulation of glycosaminoglycan degradation, highlighting its potential for preserving vascular integrity and mitigating inflammatory damage during sepsis (53, 54). Our research revealed that reduced PTGDS levels are linked to poor outcomes in sepsis and ARDS. Consequently, PTGDS might act as an indicator and be pivotal in the development of these diseases.

PID1 is involved in multiple roles, including mitochondrial morphology, boosting macromolecule metabolism, and regulating glucose homeostasis (55, 56). Our GSEA enrichment analysis results indicate that PID1 may be associated with metabolic pathways, particularly in galactose and glucose metabolism, suggests that targeting these processes may alleviate hyperglycemia-related complications, ultimately benefiting patient outcomes (57). PID1 acts as an indicator for assessing immunomodulatory treatment in sepsis and can function as a stratification tool to monitor the effectiveness of IFN-γ therapy prior to immunostimulatory intervention in sepsis (58). Furthermore, changes in PID1 are linked to reduced lung performance and heightened asthma.

The AUC was utilized to evaluate the precision of forecasting patient diagnosis or prognosis (59). At present, no research has examined the clinical significance of integrating these three genes. We created a predictive model based on three crucial genes and evaluated their diagnostic importance using ROC analysis. The AUC scores for the sepsis training and validation datasets were 0.966, 0.912, 0.941, and 0.933 in that order. Regarding ARDS, the AUC scores were 0.866 and 1, respectively. The predictive model showed high accuracy in estimating the 28-day mortality rate for sepsis and also aided in recognizing ARDS patients with a poor outlook. External datasets further validated the model’s predictive precision. These findings imply that PID1, CX3CR1, and PTGDS could be important factors in the development of ARDS and sepsis.

By conducting enrichment analysis on DEGs, we uncovered the functions and regulatory roles of various genes. We further explored the potential mechanisms and pathways of the two diseases using GO and KEGG analyses. Immune dysregulation is a characteristic of sepsis, where the initial inflammatory cascade shifts to immune suppression, leading to poor treatment outcomes and prognosis (43, 60, 61). Our GO analysis of the common differentially expressed genes in sepsis and ARDS also indicated that immune system processes such as leukocyte activation and T cell activation were downregulated. Severe impairment of oxygen and carbon dioxide exchange, a key feature of ARDS (20, 62), results from barrier damage. Additionally, we observed an increase in the ubiquitin-dependent protein degradation via the proteasome, potentially linked to the breakdown of epithelial and endothelial cell barriers. Notably, the KEGG analysis showed that upregulated pathways included platelet activation and autophagy. Excessive platelet activation can lead to thrombosis and coagulation disorders, causing multiple organ failure and ultimately high mortality in sepsis patients (3, 20). Platelet activation also exacerbates the development of ARDS, including increased neutrophil migration and endothelial barrier permeability, which intensifies inflammation and lung injury (63). In the initial phases of sepsis, autophagy helps safeguard cells by preserving mitochondrial health and inhibiting cell death (64). Nevertheless, the KEGG pathways that were suppressed encompassed antigen processing and presentation, cytotoxicity mediated by natural killer cells, and interactions between cytokines and their receptors. This resembles the findings from the GO analysis, indicating that patients with unresolved sepsis transition into an immunosuppressive phase. This phase can hinder the elimination of the initial infection, potentially causing deadly secondary infections and/or the reactivation of various dormant viruses (41, 65).

Considering that sepsis causes significant changes in the characterization and function of immune cell subsets, we further analyzed the abundance of immune cells in sepsis and ARDS groups using CIBERSORT (66). Sepsis is frequently characterized by a significant inflammatory reaction alongside immunosuppression, resulting in unfavorable treatment results and prognosis (65, 67). During the initial phase of sepsis, usually within the first 2-4 days, there is a rapid increase in white blood cells, especially neutrophils and monocytes, which is subsequently followed by a quick reduction in lymphocytes due to programmed cell death, causing immunosuppression (66). Our results align with this, indicating that sepsis patients exhibited a markedly reduced count of adaptive immune cells (CD4 and CD8 T cells) and innate immune cells (NK cells and DCs) compared to the control group, whereas monocytes, M1 macrophages, and neutrophils were significantly elevated. Moreover, compared to sepsis by itself, sepsis with ARDS showed higher infiltration of monocytes and neutrophils, as well as a decrease in CD8+ T cells and inactive NK cells. Correlation analysis of immune cells revealed that CX3CR1 expression in sepsis and ARDS patients was positively correlated with NK cell and monocyte infiltration, possibly indicating that CX3CR1 recruits these cells to inflamed tissues. PID1 was positively correlated with monocytes and DCs. PTGDS showed a positive association with activated dendritic cells and CD8 T lymphocytes, while it exhibited a negative relationship with M1 macrophages.

Subsequently, we analyzed the single-cell data of PBMCs from healthy controls, sepsis patients (both survivors and non-survivors), and sepsis-induced ARDS patients. Consistent with prior single-cell analysis results, our research demonstrated that sepsis advancement is associated with a decrease in lymphocytes, characterized by lower counts of CD8 and CD4 T cells, B cells, and NK cells (68). Additionally, we observed that as the severity of sepsis increases, there is a significant elevation in the levels of neutrophils and megakaryocytes in patients. Compared to sepsis alone, patients with sepsis-induced ARDS had fewer peripheral blood NK cells (69). In sepsis, neutrophils exhibit prolonged survival and reduced mobility, resulting in their accumulation in blood vessels. In this location, they release cytokines, reactive oxygen species (ROS), and an excess of NETs, leading to vascular inflammation and the breakdown of the endothelial barrier (70–72). Macrophages transform into the M1 phenotype during acute inflammation, secreting large amounts of pro-inflammatory molecules that drive disease progression (73). Megakaryocytes (MKs), the precursors of platelets, increase in the lungs during ARDS, releasing platelets and chromatin nets that promote inflammation and thrombosis (74, 75). Increased circulating MKs are associated with poorer sepsis outcomes (76). NK cells phenotypic changes and functional impairment are among the reasons for immunosuppression during sepsis (77). Early on, highly active NK cells exhibit cytotoxicity, capable of lysing infected cells and secreting cytotoxic proteins to combat infections (78, 79). They also secrete cytokines that activate other immune cells (80). Later, NK cells dysfunction increases the risk of secondary infections and mortality (81). In sepsis patients, NK cells numbers are significantly reduced, displaying decreased maturity, cytotoxicity, and cytokine production (77). Further investigation is needed to understand how different immune cell levels impact the outcomes of sepsis and ARDS.

Developing effective and safe drugs for sepsis and ARDS patients is of paramount importance. Our research’s focused drug screening and molecular docking findings for the trio of genes suggest that Withaferin A, SB216763, Epothilone, and Dichloroacetic Acid can interact with PTGDS, PID1, and CX3CR1, respectively, and might emerge as novel therapeutic candidates. Withaferin-A has demonstrated efficacy with diverse mechanisms, showing an anti-inflammatory impact on LPS-stimulated macrophages by reducing proinflammatory cytokines at both the transcriptional and translational stages (82). Furthermore, Withaferin-A might alleviate neutrophil-driven inflammation via various pathways, such as inhibiting inflammatory reactions and promotion apoptosis (83). SB216763, a type of Glycogen synthase kinase inhibitor, can decrease inflammatory cytokines and reduce bacterial load in Pseudomonas aeruginosa infections (84, 85). Microtubules are crucial in controlling the permeability of vascular endothelium (86). Epothilone B improved microtubule stability, significantly contributing to its anti-inflammatory impact on the management of various diseases. Epothilone’s stabilization of microtubules reduced LPS-induced endothelial cell barrier disruption *in vitro* and decreased vascular leakage and lung inflammation *in vivo* (87). In sepsis, immune cells exhibit an overactive glycolytic pathway, resulting in higher glucose usage, increased lactate levels, and intensified inflammation. Dichloroacetic acid can alleviate persistent inflammation and enhance sepsis recovery by altering the metabolic process from aerobic glycolysis to oxidative phosphorylation (88). The drugs previously discussed influence immune response, barrier integrity, or inflammation, which are typical pathological features of sepsis and ARDS. Further confirmation is needed to investigate the possible uses of these effects. These results indicate that gaining a more profound insight into the shared molecular pathways of sepsis and ARDS could be crucial for formulating effective treatments and enhancing patient recovery.

Our study has some limitations. To begin with, larger patient cohorts are needed. Using RT-qPCR, we confirmed the expression levels of three key genes in sepsis and ARDS groups relative to control groups, and the findings were consistent with the bioinformatics analysis. Although we confirmed the gene expression findings in patients and mice, larger-scale studies are needed to address the limitations of bioinformatics techniques. Due to the limitations of the datasets, some of the sepsis data we utilized were based on earlier diagnostic criteria, and the availability of clinically valuable ARDS datasets is also limited, necessitating further research in the future. Furthermore, our research involves reanalyzing existing datasets from prior publications, and we lack information on the patients’ comorbidities, treatments, and pertinent clinical details, which could introduce confounding variables into our analysis. Additionally, more studies are needed to elucidate the precise ways these genes influence the immune reaction in sepsis and ARDS. Consequently, further laboratory and animal studies, along with clinical trials, are essential to confirm our results moving forward.

## 5 Conclusion

This research is unique due to the combination of various machine learning techniques, which systematically identifying three hub genes (CX3CR1, PID1 and PTGDS) as possible biomarkers for diagnosing and predicting outcomes in sepsis and ARDS. We constructed a nomogram model, conducted survival analysis and ceRNA regulatory network, analyzed the immune microenvironment in conjunction with immune infiltration and single-cell data, and identified potential molecular compounds and corresponding molecular docking. In the end, three important genes’ differences in expression were confirmed in patient and mouse samples afflicted with sepsis and ARDS, aligning with our bioinformatics findings. This could offer new understanding into the progression of these conditions and propose fresh avenues for research aimed at preventing sepsis and ARDS.

## Data Availability

The original contributions presented in the study are included in the article/**Supplementary Material**. Further inquiries can be directed to the corresponding author.

## Supplementary material

Supplementary Figure 1 Gene enrichment analysis, WGCNA and machine learning. **(A)** GO enrichment analysis bubble graph for up-regulated DEGs. **(B)** Histogram for KEGG enrichment in up-regulated DEGs. **(C)** Cluster dendrogram displaying highly connected genes in key modules associated with sepsis of WGCNA analysis. **(D)** Visualization of the WGCNA network using a heatmap plot in sepsis. The heatmap depicts the TOM among all modules included in the analysis. The progressively darker red color represents an increasing overlap, and the light color represents a low overlap. **(E)** Dendrogram clustering of ARDS modules featuring genes with strong connectivity. **(F)** Visualization of the WGCNA network using a heatmap plot for ARDS. **(G, H)** The LASSO regression was employed to determine the minimum and lambda values for diagnostic biomarkers of sepsis and ARDS. **(I, J)** The application of Boruta was intended to find prevalent diagnostic genes of sepsis and ARDS.

Supplementary Figure 2 Relationship between CX3CR1, PID1 and PTGDS gene expression levels and clinical outcomes. **(A, C)** Expression levels of the three genes in the GSE95233 and GSE185263 datasets, where patients were categorized into death and survival groups. **(B)** Expression levels of three genes in patients with different SOFA scores in the GSE185263 dataset. **(D)** Expression of three genes in different clinical diagnosis groups in the GSE13904 dataset. **(E)** Gene expression levels of different sepsis subgroups in the GSE48080 dataset. **(F)** Gene expression levels of patients with different numbers of organ failures in the GSE63311 dataset.
