# Supplementary figures and images for: Identification and experimental validation of diagnostic and prognostic genes CX3CR1, PID1 and PTGDS in sepsis and ARDS using bulk and single-cell transcriptomic analysis and machine learning
Source: Front Immunol. 2024 Dec 23;15:1480542. doi: 10.3389/fimmu.2024.1480542 (PMC11700820; doi:10.3389/fimmu.2024.1480542)

A

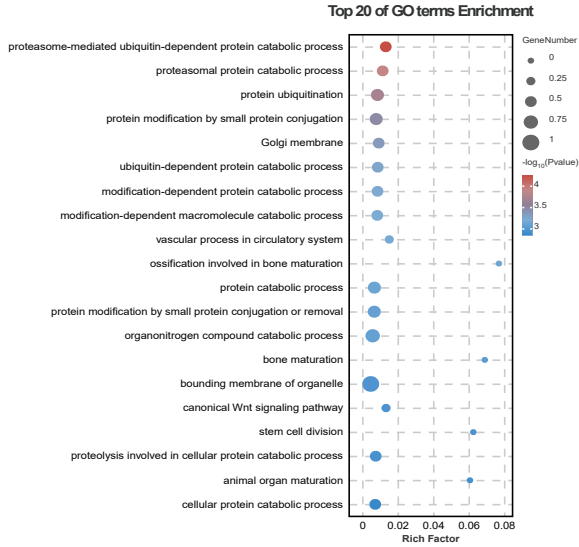

B

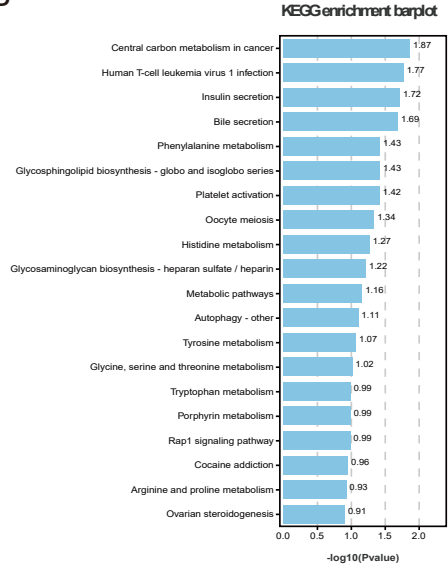

C

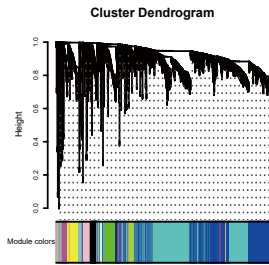

D

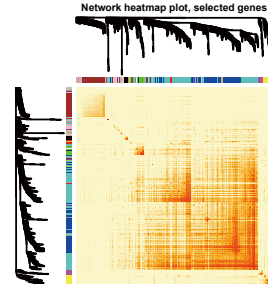

E

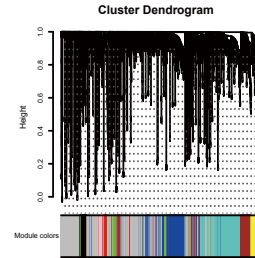

F

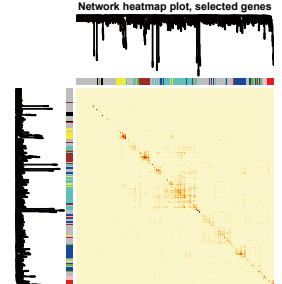

G

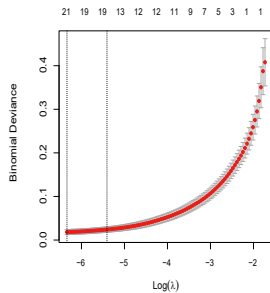

H

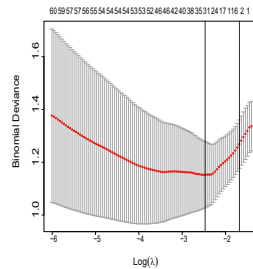

I

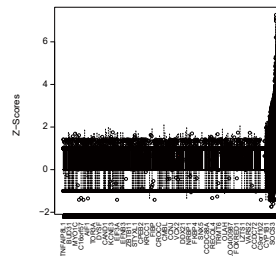

J

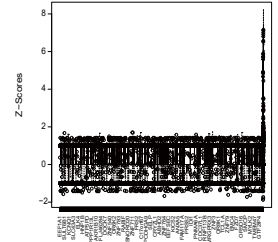

Supplement: Supplementary Figure 1 — Gene enrichment analysis, WGCNA and machine learning. (A) GO enrichment analysis bubble graph for up-regulated DEGs. (B) Histogram for KEGG enrichment in up-regulated DEGs. (C) Cluster dendrogram displaying highly connected genes in key modules associated with sepsis of WGCNA analysis. (D) Visualization of the WGCNA network using a heatmap plot in sepsis. The heatmap depicts the TOM among all modules included in the analysis. The progressively darker red color represents an increasing overlap, and the light color represents a low overlap. (E) Dendrogram clustering of ARDS modules featuring genes with strong connectivity. (F) Visualization of the WGCNA network using a heatmap plot for ARDS. (G, H) The LASSO regression was employed to determine the minimum and lambda values for diagnostic biomarkers of sepsis and ARDS. (I, J) The application of Boruta was intended to find prevalent diagnostic genes of sepsis and ARDS. [file Image1.pdf]

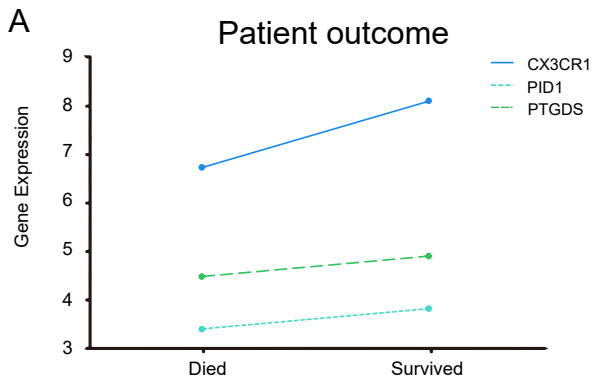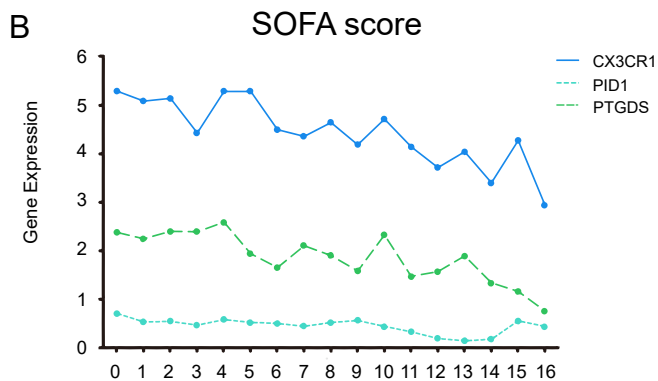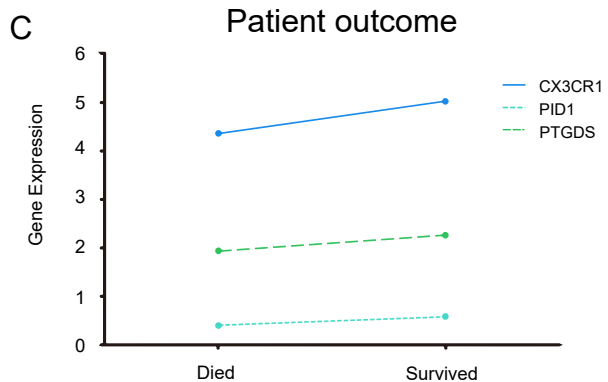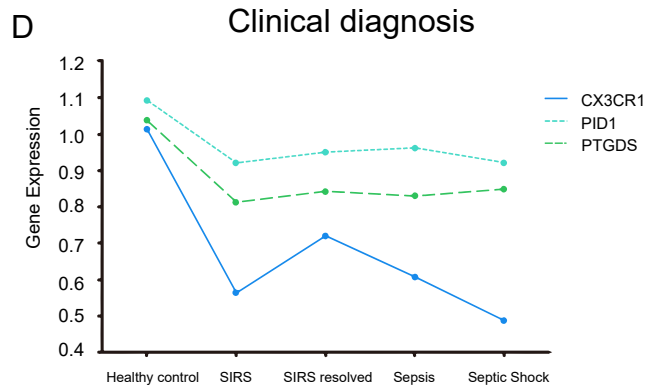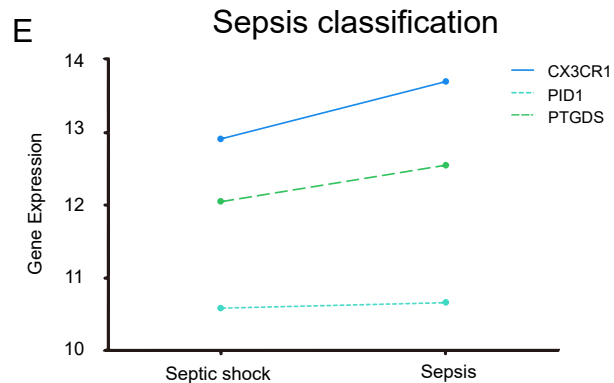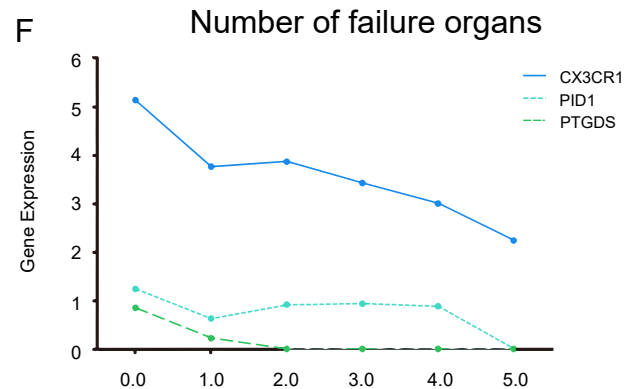

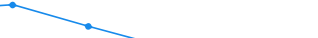

Supplement: Supplementary Figure 2 — Relationship between CX3CR1, PID1 and PTGDS gene expression levels and clinical outcomes. (A, C) Expression levels of the three genes in the GSE95233 and GSE185263 datasets, where patients were categorized into death and survival groups. (B) Expression levels of three genes in patients with different SOFA scores in the GSE185263 dataset. (D) Expression of three genes in different clinical diagnosis groups in the GSE13904 dataset. (E) Gene expression levels of different sepsis subgroups in the GSE48080 dataset. (F) Gene expression levels of patients with different numbers of organ failures in the GSE63311 dataset. [file Image2.pdf]
