# Supplementary material for: Identification and experimental validation of diagnostic and prognostic genes CX3CR1, PID1 and PTGDS in sepsis and ARDS using bulk and single-cell transcriptomic analysis and machine learning
Source: Front Immunol. 2024 Dec 23;15:1480542. doi: 10.3389/fimmu.2024.1480542 (PMC11700820; doi:10.3389/fimmu.2024.1480542)
Supplement: Supplementary file 3 [file Table1.docx]

Supplementary Material

# Table S1. The risk score in sepsis.

| id | futime (day) | fustat | risk score | risk |
| --- | --- | --- | --- | --- |
| GSM1602802 | 28 | 0 | 0.963639443 | High Risk |
| GSM1602803 | 28 | 0 | 0.606586462 | Low Risk |
| GSM1602805 | 28 | 0 | 1.494126579 | High Risk |
| GSM1602810 | 28 | 0 | 0.397947374 | Low Risk |
| GSM1602811 | 28 | 0 | 1.314972039 | High Risk |
| GSM1602812 | 28 | 0 | 1.327682396 | High Risk |
| GSM1602813 | 4 | 1 | 0.944893083 | High Risk |
| GSM1602815 | 28 | 0 | 0.846257296 | Low Risk |
| GSM1602816 | 28 | 0 | 0.565183419 | Low Risk |
| GSM1602818 | 28 | 0 | 2.211704064 | High Risk |
| GSM1602819 | 28 | 0 | 1.225012461 | High Risk |
| GSM1602820 | 28 | 0 | 0.758079482 | Low Risk |
| GSM1602823 | 17 | 1 | 1.899367648 | High Risk |
| GSM1602824 | 28 | 0 | 2.860531891 | High Risk |
| GSM1602825 | 28 | 0 | 0.662615766 | Low Risk |
| GSM1602826 | 28 | 0 | 1.241643271 | High Risk |
| id | futime (day) | fustat | riskScore | risk |
| GSM1602827 | 28 | 0 | 0.537290979 | Low Risk |
| GSM1602829 | 28 | 0 | 1.3356684 | High Risk |
| GSM1602830 | 28 | 0 | 0.755004142 | Low Risk |
| GSM1602831 | 28 | 0 | 0.487617427 | Low Risk |
| GSM1602832 | 28 | 0 | 0.783773135 | Low Risk |
| GSM1602834 | 28 | 0 | 3.01960259 | High Risk |
| GSM1602836 | 28 | 0 | 0.493667185 | Low Risk |
| GSM1602837 | 28 | 0 | 0.584507384 | Low Risk |
| GSM1602838 | 28 | 0 | 0.861221531 | Low Risk |
| GSM1602839 | 12 | 1 | 0.517279083 | Low Risk |
| GSM1602840 | 28 | 0 | 0.979018099 | High Risk |
| GSM1602841 | 28 | 0 | 0.790763795 | Low Risk |
| GSM1602842 | 28 | 0 | 0.680450406 | Low Risk |
| GSM1602843 | 28 | 0 | 0.687943894 | Low Risk |
| GSM1602844 | 7 | 1 | 2.961300723 | High Risk |
| GSM1602845 | 28 | 0 | 1.503006412 | High Risk |
| GSM1602846 | 28 | 0 | 0.455126204 | Low Risk |
| GSM1602847 | 28 | 0 | 0.548962713 | Low Risk |
| GSM1602848 | 28 | 0 | 1.007458767 | High Risk |
| id | futime (day) | fustat | riskScore | risk |
| GSM1602849 | 11 | 1 | 0.356333682 | Low Risk |
| GSM1602850 | 28 | 0 | 0.759462839 | Low Risk |
| GSM1602851 | 14 | 1 | 1.222373005 | High Risk |
| GSM1602852 | 28 | 0 | 0.63897181 | Low Risk |
| GSM1602853 | 28 | 0 | 0.71268688 | Low Risk |
| GSM1602855 | 28 | 0 | 4.600612897 | High Risk |
| GSM1602856 | 28 | 0 | 1.012724273 | High Risk |
| GSM1602857 | 28 | 0 | 1.699802981 | High Risk |
| GSM1602858 | 15 | 1 | 1.164107005 | High Risk |
| GSM1602859 | 28 | 0 | 1.72179804 | High Risk |
| GSM1602860 | 28 | 0 | 1.007999317 | High Risk |
| GSM1602861 | 28 | 0 | 0.90033287 | Low Risk |
| GSM1602863 | 1 | 1 | 1.447393465 | High Risk |
| GSM1602866 | 28 | 0 | 0.526775597 | Low Risk |
| GSM1602868 | 2 | 1 | 1.066084605 | High Risk |
| GSM1602869 | 28 | 0 | 1.252206629 | High Risk |
| GSM1602873 | 28 | 0 | 2.488960357 | High Risk |
| GSM1602878 | 28 | 0 | 0.829096527 | Low Risk |
| GSM1602879 | 28 | 0 | 1.417331875 | High Risk |
| GSM1602880 | 0 | 1 | 1.371943525 | High Risk |
| id | futime (day) | fustat | riskScore | risk |
| GSM1602881 | 28 | 0 | 0.513568622 | Low Risk |
| GSM1602882 | 28 | 0 | 0.646975744 | Low Risk |
| GSM1602883 | 28 | 0 | 2.064406624 | High Risk |
| GSM1602885 | 28 | 0 | 2.086720907 | High Risk |
| GSM1602886 | 28 | 0 | 1.914829102 | High Risk |
| GSM1602888 | 8 | 0 | 0.453199246 | Low Risk |
| GSM1602889 | 28 | 0 | 0.883316149 | Low Risk |
| GSM1602902 | 28 | 0 | 1.700483551 | High Risk |
| GSM1602903 | 17 | 1 | 1.861047058 | High Risk |
| GSM1602906 | 28 | 0 | 0.643223884 | Low Risk |
| GSM1602907 | 28 | 0 | 0.675059303 | Low Risk |
| GSM1602908 | 0 | 1 | 2.936429474 | High Risk |
| GSM1602912 | 8 | 1 | 2.835960628 | High Risk |
| GSM1602921 | 28 | 0 | 0.730836881 | Low Risk |
| GSM1602922 | 28 | 0 | 1.130265268 | High Risk |
| GSM1602923 | 1 | 1 | 5.353958113 | High Risk |
| GSM1602924 | 28 | 0 | 1.482384895 | High Risk |
| GSM1602925 | 28 | 0 | 1.020718594 | High Risk |
| GSM1602927 | 0 | 1 | 2.120068452 | High Risk |
| id | futime (day) | fustat | riskScore | risk |
| GSM1602928 | 14 | 1 | 1.541715539 | High Risk |
| GSM1602929 | 28 | 0 | 1.301771559 | High Risk |
| GSM1602930 | 28 | 0 | 0.842125457 | Low Risk |
| GSM1602931 | 20 | 1 | 0.724512239 | Low Risk |
| GSM1602932 | 15 | 1 | 2.425272956 | High Risk |
| GSM1602933 | 28 | 0 | 0.99343347 | High Risk |
| GSM1602934 | 22 | 1 | 1.689548825 | High Risk |
| GSM1602935 | 28 | 0 | 0.993299177 | High Risk |
| GSM1602936 | 28 | 0 | 1.011961254 | High Risk |
| GSM1602937 | 28 | 0 | 0.515752121 | Low Risk |
| GSM1602940 | 28 | 0 | 1.06323276 | High Risk |
| GSM1602942 | 28 | 1 | 0.925763711 | Low Risk |
| GSM1602943 | 16 | 1 | 0.653147288 | Low Risk |
| GSM1602944 | 28 | 0 | 1.305495327 | High Risk |
| GSM1602945 | 28 | 0 | 0.586830027 | Low Risk |
| GSM1602946 | 8 | 1 | 0.968650751 | High Risk |
| GSM1602950 | 28 | 0 | 0.839988127 | Low Risk |
| GSM1602952 | 28 | 0 | 0.922971263 | Low Risk |
| GSM1602953 | 28 | 0 | 1.594633974 | High Risk |
| GSM1602958 | 28 | 0 | 1.129537308 | High Risk |
| id | futime (day) | fustat | riskScore | risk |
| GSM1602968 | 28 | 0 | 0.743147882 | Low Risk |
| GSM1602973 | 28 | 0 | 0.453390901 | Low Risk |
| GSM1602974 | 28 | 0 | 1.599100984 | High Risk |
| GSM1602975 | 28 | 0 | 0.574774911 | Low Risk |
| GSM1602976 | 28 | 0 | 1.326523823 | High Risk |
| GSM1691861 | 6 | 1 | 1.473481379 | High Risk |
| GSM1691863 | 28 | 0 | 2.002883982 | High Risk |
| GSM1691864 | 12 | 1 | 0.859268549 | Low Risk |
| GSM1691865 | 28 | 0 | 1.469672292 | High Risk |
| GSM1691866 | 28 | 0 | 0.683999458 | Low Risk |
| GSM1691867 | 28 | 0 | 1.068552255 | High Risk |
| GSM1691868 | 7 | 1 | 0.418473571 | Low Risk |
| GSM1691871 | 28 | 0 | 1.484932797 | High Risk |
| GSM1691873 | 28 | 0 | 0.570567955 | Low Risk |
| GSM1691874 | 28 | 0 | 4.567451566 | High Risk |
| GSM1691875 | 28 | 0 | 1.315743718 | High Risk |
| GSM1691877 | 28 | 0 | 1.833518677 | High Risk |
| GSM1691878 | 28 | 0 | 0.733009238 | Low Risk |
| GSM1691880 | 11 | 1 | 2.90145119 | High Risk |
| id | futime (day) | fustat | riskScore | risk |
| GSM1691882 | 28 | 0 | 1.832848309 | High Risk |
| GSM1691883 | 28 | 0 | 0.508972229 | Low Risk |
| GSM1691884 | 0 | 1 | 0.734924546 | Low Risk |
| GSM1691885 | 28 | 0 | 0.430809655 | Low Risk |
| GSM1691886 | 28 | 0 | 0.651614568 | Low Risk |
| GSM1691890 | 28 | 0 | 0.670256456 | Low Risk |
| GSM1691893 | 28 | 0 | 1.024332373 | High Risk |
| GSM1691895 | 28 | 0 | 0.949786558 | High Risk |
| GSM1691896 | 28 | 0 | 0.773980511 | Low Risk |
| GSM1691897 | 28 | 0 | 0.510630249 | Low Risk |
| GSM1691898 | 28 | 0 | 1.898818841 | High Risk |
| GSM1691900 | 13 | 1 | 1.154254372 | High Risk |
| GSM1691901 | 28 | 0 | 1.062148267 | High Risk |
| GSM1691903 | 28 | 0 | 0.913870653 | Low Risk |
| GSM1691904 | 28 | 0 | 0.713349332 | Low Risk |
| GSM1691905 | 28 | 0 | 0.540074281 | Low Risk |
| GSM1691909 | 28 | 0 | 0.681322799 | Low Risk |
| GSM1691911 | 28 | 0 | 0.448862814 | Low Risk |
| GSM1691915 | 28 | 0 | 1.159610972 | High Risk |
| GSM1691918 | 28 | 0 | 1.254681669 | High Risk |
| id | futime (day) | fustat | riskScore | risk |
| GSM1691923 | 28 | 0 | 0.247783262 | Low Risk |
| GSM1691925 | 28 | 0 | 0.60745224 | Low Risk |
| GSM1691927 | 28 | 0 | 1.893904503 | High Risk |
| GSM1691928 | 28 | 0 | 0.871645047 | Low Risk |
| GSM1691929 | 28 | 0 | 0.678190419 | Low Risk |
| GSM1691932 | 4 | 1 | 1.51393148 | High Risk |
| GSM1691933 | 28 | 0 | 2.086356521 | High Risk |
| GSM1691934 | 28 | 0 | 0.770193786 | Low Risk |
| GSM1691935 | 15 | 1 | 0.85305828 | Low Risk |
| GSM1691937 | 7 | 1 | 2.1991503 | High Risk |
| GSM1691938 | 28 | 0 | 0.654743611 | Low Risk |
| GSM1691941 | 28 | 0 | 2.380208661 | High Risk |
| GSM1691942 | 28 | 0 | 2.896042172 | High Risk |
| GSM1691943 | 12 | 1 | 1.321800408 | High Risk |
| GSM1691944 | 28 | 0 | 0.767419831 | Low Risk |
| GSM1691946 | 8 | 1 | 0.772925028 | Low Risk |
| GSM1691947 | 28 | 0 | 0.687315053 | Low Risk |
| GSM1691953 | 28 | 0 | 0.508174812 | Low Risk |
| GSM1691954 | 28 | 0 | 1.106359298 | High Risk |
| id | futime (day) | fustat | riskScore | risk |
| GSM1691957 | 28 | 0 | 1.203009495 | High Risk |
| GSM1691959 | 28 | 0 | 0.57742574 | Low Risk |
| GSM1691960 | 28 | 0 | 0.423655712 | Low Risk |
| GSM1691961 | 28 | 0 | 0.851028535 | Low Risk |
| GSM1691962 | 28 | 0 | 0.67782947 | Low Risk |
| GSM1691963 | 28 | 0 | 1.354360666 | High Risk |
| GSM1691965 | 15 | 1 | 0.756107777 | Low Risk |
| GSM1691966 | 8 | 1 | 2.037216533 | High Risk |
| GSM1691968 | 28 | 0 | 0.998646596 | High Risk |
| GSM1691969 | 0 | 1 | 2.987257495 | High Risk |
| GSM1691970 | 28 | 0 | 1.659278946 | High Risk |
| GSM1691973 | 28 | 0 | 0.327663229 | Low Risk |
| GSM1691977 | 28 | 0 | 1.253580659 | High Risk |
| GSM1691980 | 28 | 0 | 1.027615986 | High Risk |
| GSM1691981 | 0 | 1 | 2.472543238 | High Risk |
| GSM1691983 | 28 | 0 | 2.196187292 | High Risk |
| GSM1691984 | 2 | 1 | 3.258933566 | High Risk |
| GSM1691985 | 13 | 1 | 2.616986785 | High Risk |
| GSM1691986 | 28 | 0 | 0.912214719 | Low Risk |
| id | futime (day) | fustat | riskScore | risk |
| GSM1691987 | 28 | 0 | 2.309654846 | High Risk |
| GSM1691989 | 28 | 0 | 0.590238522 | Low Risk |
| GSM1691990 | 28 | 0 | 0.396206155 | Low Risk |
| GSM1691991 | 5 | 1 | 0.993727184 | High Risk |
| GSM1691992 | 14 | 1 | 1.23019158 | High Risk |
| GSM1691993 | 28 | 0 | 0.525821974 | Low Risk |
| GSM1691994 | 28 | 0 | 0.789869182 | Low Risk |
| GSM1691995 | 28 | 0 | 1.829762102 | High Risk |
| GSM1691997 | 28 | 0 | 0.529599603 | Low Risk |
| GSM1692000 | 28 | 0 | 0.427364368 | Low Risk |
| GSM1692002 | 28 | 0 | 1.085515964 | High Risk |
| GSM1692004 | 6 | 1 | 0.741126444 | Low Risk |
| GSM1692005 | 28 | 0 | 0.56872351 | Low Risk |
| GSM1692006 | 3 | 1 | 1.332154782 | High Risk |
| GSM1692007 | 28 | 0 | 0.90743648 | Low Risk |
| GSM1692008 | 28 | 0 | 0.725794209 | Low Risk |
| GSM1692009 | 28 | 0 | 1.113969402 | High Risk |
| GSM1692011 | 2 | 1 | 2.068247727 | High Risk |
| GSM1692012 | 28 | 0 | 1.028779816 | High Risk |
| id | futime (day) | fustat | riskScore | risk |
| GSM1692016 | 28 | 0 | 3.510167852 | High Risk |
| GSM1692017 | 2 | 1 | 0.781548108 | Low Risk |
| GSM1692019 | 28 | 0 | 0.744236718 | Low Risk |
| GSM1692022 | 28 | 0 | 1.900096814 | High Risk |
| GSM1692023 | 28 | 0 | 0.74535296 | Low Risk |
| GSM1692024 | 3 | 1 | 1.70552477 | High Risk |
| GSM1692025 | 28 | 0 | 0.357999231 | Low Risk |
| GSM1692029 | 3 | 1 | 1.889407853 | High Risk |
| GSM1692030 | 28 | 0 | 0.916526836 | Low Risk |
| GSM1692032 | 28 | 0 | 0.763098854 | Low Risk |
| GSM1692033 | 28 | 0 | 0.317614119 | Low Risk |
| GSM1692034 | 28 | 0 | 1.057633418 | High Risk |
| GSM1692035 | 28 | 0 | 0.517116448 | Low Risk |
| GSM1692038 | 12 | 1 | 2.442175956 | High Risk |
| GSM1692040 | 28 | 0 | 0.623520656 | Low Risk |
| GSM1692041 | 28 | 0 | 0.961031497 | High Risk |
| GSM1692042 | 28 | 0 | 0.803950098 | Low Risk |
| GSM1692043 | 1 | 1 | 1.670422755 | High Risk |
| GSM1692045 | 28 | 0 | 1.0771129 | High Risk |
| GSM1692047 | 1 | 1 | 1.702150971 | High Risk |
| id | futime (day) | fustat | riskScore | risk |
| GSM1692050 | 28 | 0 | 1.945707254 | High Risk |
| GSM1692051 | 28 | 0 | 0.761690043 | Low Risk |
| GSM1692052 | 28 | 0 | 0.562689528 | Low Risk |
| GSM1692055 | 28 | 0 | 2.466058417 | High Risk |
| GSM1692056 | 1 | 1 | 3.437508105 | High Risk |
| GSM1692058 | 28 | 0 | 0.67489161 | Low Risk |
| GSM1692059 | 28 | 0 | 1.575940207 | High Risk |
| GSM1692060 | 28 | 0 | 2.097030338 | High Risk |
| GSM1692061 | 28 | 0 | 0.751955325 | Low Risk |
| GSM1692063 | 28 | 0 | 1.021167778 | High Risk |
| GSM1692064 | 6 | 1 | 0.624301603 | Low Risk |
| GSM1692065 | 28 | 0 | 0.811790328 | Low Risk |
| GSM1692066 | 28 | 0 | 1.058891991 | High Risk |
| GSM1692068 | 28 | 0 | 0.840235223 | Low Risk |
| GSM1692071 | 1 | 1 | 1.247184007 | High Risk |
| GSM1692073 | 28 | 0 | 1.293759494 | High Risk |
| GSM1692074 | 28 | 0 | 0.629648547 | Low Risk |
| GSM1692077 | 28 | 0 | 0.549339606 | Low Risk |
| GSM1692079 | 1 | 1 | 3.064980806 | High Risk |
| id | futime (day) | fustat | riskScore | risk |
| GSM1692081 | 28 | 0 | 0.769469163 | Low Risk |
| GSM1692084 | 28 | 0 | 1.450128889 | High Risk |
| GSM1692086 | 28 | 0 | 2.043365688 | High Risk |
| GSM1692087 | 28 | 0 | 0.721008679 | Low Risk |
| GSM1692089 | 28 | 0 | 0.509082364 | Low Risk |
| GSM1692092 | 5 | 1 | 1.707926482 | High Risk |
| GSM1692093 | 28 | 0 | 0.596694104 | Low Risk |
| GSM1692094 | 28 | 0 | 0.614832801 | Low Risk |
| GSM1692096 | 28 | 0 | 1.353293333 | High Risk |
| GSM1692099 | 28 | 0 | 3.75338703 | High Risk |
| GSM1692100 | 5 | 1 | 1.410559565 | High Risk |
| GSM1692101 | 28 | 0 | 0.476322881 | Low Risk |
| GSM1692103 | 28 | 0 | 1.790328701 | High Risk |
| GSM1692105 | 0 | 1 | 0.835664114 | Low Risk |
| GSM1692107 | 28 | 0 | 0.526909728 | Low Risk |
| GSM1692108 | 28 | 0 | 1.562687716 | High Risk |
| GSM1692109 | 28 | 0 | 0.501253308 | Low Risk |
| GSM1692112 | 3 | 1 | 0.678677185 | Low Risk |
| GSM1692114 | 12 | 1 | 0.952551874 | High Risk |
| GSM1692115 | 28 | 0 | 1.64269644 | High Risk |
| id | futime (day) | fustat | riskScore | risk |
| GSM1692116 | 28 | 0 | 0.773046732 | Low Risk |
| GSM1692117 | 28 | 0 | 0.821504198 | Low Risk |
| GSM1692118 | 28 | 0 | 1.936169358 | High Risk |
| GSM1692119 | 6 | 1 | 1.576976562 | High Risk |
| GSM1692121 | 28 | 0 | 0.417318803 | Low Risk |
| GSM1692123 | 28 | 0 | 1.916625082 | High Risk |
| GSM1692124 | 14 | 1 | 0.616616769 | Low Risk |
| GSM1692125 | 28 | 0 | 0.983048518 | High Risk |
| GSM1692126 | 28 | 0 | 1.018433756 | High Risk |
| GSM1692127 | 28 | 0 | 0.42213775 | Low Risk |
| GSM1692130 | 28 | 0 | 0.648625485 | Low Risk |
| GSM1692132 | 28 | 0 | 0.798371417 | Low Risk |
| GSM1692133 | 28 | 0 | 0.624345127 | Low Risk |
| GSM1692136 | 28 | 0 | 0.952640704 | High Risk |
| GSM1692137 | 28 | 0 | 0.803604462 | Low Risk |
| GSM1692138 | 28 | 0 | 1.06855879 | High Risk |
| GSM1692139 | 28 | 0 | 1.584010061 | High Risk |
| GSM1692140 | 28 | 0 | 2.351742159 | High Risk |
| GSM1692141 | 28 | 0 | 0.6995917 | Low Risk |
| id | futime (day) | fustat | riskScore | risk |
| GSM1692142 | 28 | 0 | 0.572808288 | Low Risk |
| GSM1692143 | 28 | 0 | 1.518209658 | High Risk |
| GSM1692144 | 4 | 1 | 1.333690443 | High Risk |
| GSM1692145 | 28 | 0 | 0.93566577 | High Risk |
| GSM1692146 | 3 | 1 | 1.857361564 | High Risk |
| GSM1692148 | 10 | 1 | 1.216200584 | High Risk |
| GSM1692149 | 28 | 1 | 1.65047207 | High Risk |
| GSM1692151 | 15 | 1 | 0.838131173 | Low Risk |
| GSM1692153 | 28 | 0 | 1.269144935 | High Risk |
| GSM1692156 | 28 | 0 | 1.972724172 | High Risk |
| GSM1692157 | 28 | 0 | 1.24441762 | High Risk |
| GSM1692158 | 28 | 0 | 0.665522865 | Low Risk |
| GSM1692160 | 2 | 1 | 3.881869596 | High Risk |
| GSM1692162 | 28 | 0 | 0.696707863 | Low Risk |
| GSM1692163 | 18 | 1 | 0.82218017 | Low Risk |
| GSM1692165 | 28 | 0 | 0.513360623 | Low Risk |
| GSM1692166 | 28 | 0 | 1.932002394 | High Risk |
| GSM1692167 | 28 | 0 | 0.48579264 | Low Risk |
| GSM1692168 | 28 | 0 | 0.679327364 | Low Risk |
| GSM1692169 | 12 | 1 | 0.928796636 | Low Risk |
| id | futime (day) | fustat | riskScore | risk |
| GSM1692170 | 7 | 1 | 1.55271015 | High Risk |
| GSM1692172 | 28 | 0 | 1.135297266 | High Risk |
| GSM1692174 | 28 | 0 | 1.572937685 | High Risk |
| GSM1692175 | 4 | 1 | 1.337632803 | High Risk |
| GSM1692181 | 28 | 0 | 0.809452781 | Low Risk |
| GSM1692182 | 3 | 1 | 0.495040947 | Low Risk |
| GSM1692184 | 1 | 1 | 1.068448366 | High Risk |
| GSM1692186 | 2 | 1 | 1.057953973 | High Risk |
| GSM1692187 | 28 | 0 | 0.364752805 | Low Risk |
| GSM1692188 | 28 | 0 | 0.62934428 | Low Risk |
| GSM1692190 | 3 | 1 | 0.824798597 | Low Risk |
| GSM1692191 | 28 | 0 | 1.511230537 | High Risk |
| GSM1692192 | 28 | 0 | 1.487659862 | High Risk |
| GSM1692193 | 28 | 0 | 0.796216832 | Low Risk |
| GSM1692196 | 28 | 0 | 1.56432684 | High Risk |
| GSM1692197 | 28 | 0 | 0.390908958 | Low Risk |
| GSM1692198 | 28 | 0 | 2.045131847 | High Risk |
| GSM1692199 | 28 | 0 | 0.686743801 | Low Risk |
| GSM1692201 | 28 | 0 | 1.206782909 | High Risk |
| id | futime (day) | fustat | riskScore | risk |
| GSM1692202 | 28 | 0 | 0.838010584 | Low Risk |
| GSM1692203 | 28 | 0 | 1.772775232 | High Risk |
| GSM1692204 | 28 | 0 | 0.605424108 | Low Risk |
| GSM1692205 | 23 | 1 | 1.356481527 | High Risk |
| GSM1692206 | 28 | 0 | 0.764295695 | Low Risk |
| GSM1692207 | 28 | 0 | 0.69044406 | Low Risk |
| GSM1692209 | 6 | 1 | 0.569545436 | Low Risk |
| GSM1692211 | 12 | 1 | 0.766861806 | Low Risk |
| GSM1692212 | 28 | 0 | 1.855804463 | High Risk |
| GSM1692213 | 28 | 0 | 0.837147988 | Low Risk |
| GSM1692214 | 7 | 1 | 1.412143578 | High Risk |
| GSM1692216 | 28 | 0 | 0.704201526 | Low Risk |
| GSM1692217 | 28 | 0 | 1.376713404 | High Risk |
| GSM1692221 | 28 | 0 | 1.332914425 | High Risk |
| GSM1692222 | 28 | 0 | 0.569503175 | Low Risk |
| GSM1692223 | 14 | 1 | 0.558990392 | Low Risk |
| GSM1692224 | 27 | 1 | 2.441055819 | High Risk |
| GSM1692225 | 28 | 0 | 1.302106943 | High Risk |
| GSM1692226 | 28 | 0 | 1.443414385 | High Risk |
| GSM1692227 | 28 | 0 | 0.54857256 | Low Risk |
| id | futime (day) | fustat | riskScore | risk |
| GSM1692228 | 28 | 0 | 0.714564739 | Low Risk |
| GSM1692230 | 28 | 0 | 0.852737625 | Low Risk |
| GSM1692232 | 28 | 0 | 1.030188903 | High Risk |
| GSM1692238 | 28 | 0 | 1.312641203 | High Risk |
| GSM1692240 | 28 | 0 | 1.081518119 | High Risk |
| GSM1692241 | 16 | 1 | 0.974477961 | High Risk |
| GSM1692242 | 28 | 0 | 1.394839958 | High Risk |
| GSM1692243 | 28 | 0 | 1.765537526 | High Risk |
| GSM1692244 | 7 | 1 | 0.386152689 | Low Risk |
| GSM1692245 | 28 | 0 | 0.490293709 | Low Risk |
| GSM1692247 | 28 | 0 | 0.409157807 | Low Risk |
| GSM1692251 | 3 | 1 | 2.963627952 | High Risk |
| GSM1692255 | 28 | 0 | 0.746178231 | Low Risk |
| GSM1692257 | 28 | 0 | 1.209842753 | High Risk |
| GSM1692258 | 28 | 0 | 0.535576792 | Low Risk |
| GSM1692259 | 28 | 0 | 0.347532315 | Low Risk |
| GSM1692260 | 28 | 0 | 1.095282529 | High Risk |
| GSM1692261 | 28 | 0 | 1.080236663 | High Risk |
| GSM1692264 | 10 | 1 | 1.100046664 | High Risk |
| id | futime (day) | fustat | riskScore | risk |
| GSM1692266 | 28 | 0 | 0.801739258 | Low Risk |
| GSM1692268 | 0 | 1 | 0.975439506 | High Risk |
| GSM1692269 | 28 | 0 | 0.621652072 | Low Risk |
| GSM1692271 | 10 | 1 | 1.002852666 | High Risk |
| GSM1692272 | 28 | 0 | 0.406538806 | Low Risk |
| GSM1692274 | 28 | 0 | 1.639539197 | High Risk |
| GSM1692277 | 28 | 0 | 0.929871276 | Low Risk |
| GSM1692278 | 28 | 0 | 0.808136244 | Low Risk |
| GSM1692279 | 2 | 1 | 1.346662719 | High Risk |
| GSM1692284 | 28 | 0 | 0.837695512 | Low Risk |
| GSM1692286 | 28 | 0 | 0.756115989 | Low Risk |
| GSM1692289 | 28 | 0 | 1.452752303 | High Risk |
| GSM1692291 | 28 | 0 | 0.72496671 | Low Risk |
| GSM1692292 | 0 | 1 | 0.743484173 | Low Risk |
| GSM1692294 | 28 | 0 | 0.627333797 | Low Risk |
| GSM1692296 | 28 | 0 | 0.386168314 | Low Risk |
| GSM1692298 | 28 | 0 | 0.664383452 | Low Risk |
| GSM1692299 | 21 | 1 | 1.683264367 | High Risk |
| GSM1692300 | 28 | 0 | 0.590085073 | Low Risk |
| GSM1692301 | 28 | 0 | 1.54098049 | High Risk |
| id | futime (day) | fustat | riskScore | risk |
| GSM1692302 | 28 | 0 | 1.072666002 | High Risk |
| GSM1692303 | 28 | 0 | 0.733884758 | Low Risk |
| GSM1692304 | 28 | 0 | 0.933099983 | Low Risk |
| GSM1692305 | 28 | 0 | 2.86799634 | High Risk |
| GSM1692308 | 28 | 0 | 1.907235113 | High Risk |
| GSM1692309 | 28 | 0 | 0.4960344 | Low Risk |
| GSM1692311 | 11 | 1 | 1.086712046 | High Risk |
| GSM1692312 | 0 | 1 | 4.094170399 | High Risk |
| GSM1692315 | 28 | 0 | 0.603806561 | Low Risk |
| GSM1692316 | 1 | 1 | 2.338438725 | High Risk |
| GSM1692317 | 28 | 0 | 0.630378089 | Low Risk |
| GSM1692318 | 1 | 1 | 0.644156299 | Low Risk |
| GSM1692319 | 28 | 0 | 0.618788972 | Low Risk |
| GSM1692322 | 28 | 0 | 0.924626926 | Low Risk |
| GSM1692326 | 25 | 1 | 0.440082574 | Low Risk |
| GSM1692327 | 28 | 0 | 1.057511522 | High Risk |
| GSM1692329 | 5 | 1 | 4.290354813 | High Risk |
| GSM1692330 | 28 | 0 | 0.708876083 | Low Risk |
| GSM1692331 | 2 | 1 | 1.790622557 | High Risk |
| id | futime (day) | fustat | riskScore | risk |
| GSM1692332 | 28 | 0 | 1.193332591 | High Risk |
| GSM1692335 | 28 | 0 | 0.759726564 | Low Risk |
| GSM1692336 | 1 | 1 | 0.755978125 | Low Risk |
| GSM1692337 | 28 | 0 | 1.263868735 | High Risk |
| GSM1692338 | 28 | 0 | 0.441974236 | Low Risk |
| GSM1692340 | 2 | 1 | 0.712015551 | Low Risk |
| GSM1692343 | 8 | 1 | 0.90481527 | Low Risk |
| GSM1692347 | 28 | 0 | 1.241560305 | High Risk |
| GSM1692348 | 28 | 0 | 0.866521119 | Low Risk |
| GSM1692350 | 0 | 1 | 1.60948519 | High Risk |
| GSM1692352 | 8 | 1 | 0.864131108 | Low Risk |
| GSM1692355 | 28 | 0 | 0.936596872 | High Risk |
| GSM1692356 | 28 | 0 | 1.037350182 | High Risk |
| GSM1692357 | 28 | 0 | 0.584716805 | Low Risk |
| GSM1692360 | 28 | 0 | 0.749591392 | Low Risk |
| GSM1692361 | 28 | 0 | 1.439198511 | High Risk |
| GSM1692362 | 28 | 0 | 1.116651162 | High Risk |
| GSM1692364 | 28 | 0 | 0.994094274 | High Risk |
| GSM1692366 | 28 | 0 | 1.949142676 | High Risk |
| GSM1692369 | 15 | 1 | 0.773505795 | Low Risk |
| id | futime (day) | fustat | riskScore | risk |
| GSM1692373 | 28 | 0 | 0.633332666 | Low Risk |
| GSM1692374 | 28 | 0 | 0.901878434 | Low Risk |
| GSM1692375 | 28 | 0 | 1.836894533 | High Risk |
| GSM1692376 | 11 | 1 | 1.139259781 | High Risk |
| GSM1692377 | 2 | 1 | 0.533710118 | Low Risk |
| GSM1692379 | 5 | 0 | 2.56978627 | High Risk |
| GSM1692381 | 28 | 0 | 0.957459956 | High Risk |
| GSM1692384 | 28 | 0 | 0.614996596 | Low Risk |
| GSM1692386 | 28 | 0 | 0.902881272 | Low Risk |
| GSM1692387 | 1 | 1 | 2.710220303 | High Risk |
| GSM1692388 | 18 | 1 | 1.375719008 | High Risk |
| GSM1692389 | 19 | 1 | 0.449425564 | Low Risk |
| GSM1692391 | 28 | 0 | 1.392004982 | High Risk |
| GSM1692392 | 28 | 0 | 0.428609635 | Low Risk |
| GSM1692393 | 28 | 0 | 0.870893534 | Low Risk |
| GSM1692394 | 1 | 1 | 0.877377198 | Low Risk |
| GSM1692395 | 28 | 0 | 0.904954574 | Low Risk |
| GSM1692397 | 28 | 0 | 0.750673803 | Low Risk |
| GSM1692399 | 28 | 0 | 3.956934925 | High Risk |
| id | futime (day) | fustat | riskScore | risk |
| GSM1692400 | 28 | 0 | 0.868330688 | Low Risk |
| GSM1692404 | 28 | 0 | 0.52272054 | Low Risk |
| GSM1692406 | 28 | 0 | 0.416642051 | Low Risk |
| GSM1692407 | 28 | 0 | 0.498137869 | Low Risk |
| GSM1692408 | 28 | 0 | 1.037205566 | High Risk |
| GSM1692410 | 28 | 0 | 1.359706102 | High Risk |
| GSM1692412 | 28 | 0 | 2.520935371 | High Risk |
| GSM1692413 | 28 | 0 | 0.632766204 | Low Risk |
| GSM1692414 | 28 | 0 | 1.551508896 | High Risk |
| GSM1692415 | 28 | 0 | 0.531771346 | Low Risk |
| GSM1692416 | 28 | 0 | 1.357391959 | High Risk |
| GSM1692417 | 28 | 0 | 0.440801261 | Low Risk |
| GSM1692418 | 28 | 0 | 0.88030431 | Low Risk |
| GSM1692420 | 28 | 0 | 0.800348238 | Low Risk |
| GSM1692421 | 28 | 0 | 1.481157112 | High Risk |
| GSM1692422 | 28 | 0 | 0.657763587 | Low Risk |
| GSM1692423 | 28 | 0 | 0.328038382 | Low Risk |
| GSM1692424 | 28 | 0 | 0.938401768 | High Risk |
| GSM1692425 | 28 | 0 | 1.211678619 | High Risk |
| GSM1692427 | 28 | 0 | 0.803752402 | Low Risk |
| id | futime (day) | fustat | riskScore | risk |
| GSM1692428 | 28 | 0 | 0.56689004 | Low Risk |
| GSM1692429 | 28 | 0 | 0.925149797 | Low Risk |
| GSM1692430 | 28 | 0 | 0.882963139 | Low Risk |
| GSM1692436 | 28 | 0 | 0.842179649 | Low Risk |
| GSM1692437 | 28 | 0 | 0.689185038 | Low Risk |
| GSM1692438 | 28 | 0 | 1.048231533 | High Risk |
| GSM1692439 | 28 | 0 | 0.884813023 | Low Risk |
| GSM1692440 | 28 | 0 | 0.800377447 | Low Risk |
| GSM1692441 | 28 | 0 | 0.560955405 | Low Risk |
| GSM1692443 | 8 | 1 | 1.896576744 | High Risk |
| GSM1692445 | 28 | 0 | 0.434874583 | Low Risk |
| GSM1692446 | 28 | 0 | 1.318860926 | High Risk |
| GSM1692448 | 28 | 0 | 1.015504353 | High Risk |
| GSM1692452 | 28 | 0 | 0.829218132 | Low Risk |
| GSM1692453 | 28 | 0 | 1.506556375 | High Risk |
| GSM1692454 | 17 | 1 | 1.299764362 | High Risk |
| GSM1692455 | 28 | 0 | 0.501285701 | Low Risk |
| GSM1692458 | 28 | 0 | 1.7625275 | High Risk |
| GSM1692459 | 20 | 1 | 1.473753611 | High Risk |
| id | futime (day) | fustat | riskScore | risk |
| GSM1692461 | 28 | 0 | 0.681479582 | Low Risk |
| GSM1692465 | 28 | 0 | 2.961647034 | High Risk |
| GSM1692466 | 28 | 0 | 0.874158994 | Low Risk |
| GSM1692467 | 1 | 1 | 4.229584006 | High Risk |
| GSM1692478 | 13 | 1 | 1.470107268 | High Risk |
| GSM1692482 | 28 | 0 | 1.46292293 | High Risk |
| GSM1692484 | 28 | 0 | 1.751753959 | High Risk |
| GSM1692488 | 28 | 0 | 2.085669304 | High Risk |
| GSM1692489 | 28 | 0 | 0.770210285 | Low Risk |
| GSM1692491 | 28 | 0 | 0.93286608 | Low Risk |
| GSM1692492 | 28 | 0 | 0.699973739 | Low Risk |
| GSM1692493 | 28 | 0 | 0.696255795 | Low Risk |
| GSM1692494 | 28 | 0 | 0.599266108 | Low Risk |
| GSM1692498 | 28 | 0 | 0.58247462 | Low Risk |
| GSM1692499 | 28 | 0 | 1.549786804 | High Risk |
| GSM1692501 | 28 | 0 | 0.696554789 | Low Risk |

# The risk score for each sample was calculated using the formula: Risk score = (-0.38829984) × CX3CR1 expression + (0.12871648) × PID1 expression+ (-0.08360305) × PTGDS expression.

Table S2. Correlations between three genes and key clinical outcomes.

|  |  | |  | | | |
| --- | --- | --- | --- | --- | --- | --- |
|  | |  | | CX3CR1 | PID1 | PTGDS |
| Death outcome | | Coefficient | | -0.450 | -0.455 | -0.554 |
|  |  | *p* value | | *p<0.01* | *p<0.01* | *p<0.01* |
| SOFA score 24h  post admisssion | | Coefficient | | -0.261 | -0.239 | -0.149 |
|  |  | *p* value | | *p<0.01* | *p<0.01* | *p<0.01* |
| Failure organ counts | | Coefficient | | -0.476 | -0.232 | -0.324 |
|  |  | *p* value | | *p<0.01* | *0.057* | *p<0.01* |
|  | |  | | |  |  |
